# Retinitis Pigmentosa: A Review of Its Pathogenesis and Emerging Molecular and Cellular Therapies

**DOI:** 10.3390/vision10040083

**Published:** 2026-10-14

**Authors:** Nicolas Tuli, Moath Baeshen, Mohamed Echbicheb, Merve Kulbay, Anfisa Ayalon, Shigufa Kahn Ali, Cynthia X. Qian

**Affiliations:** 1 Faculty of Medicine and Health Sciences, McGill University, Montreal, QC H3G 2M1, Canada; nicolas.tuli@mail.mcgill.ca; 2 Department of Ophthalmology & Visual Sciences, McGill University, Montreal, QC H4A 3S5, Canada; moath.baeshen@mail.mcgill.ca (M.B.); merve.kulbay@mail.mcgill.ca (M.K.); anfisa.ayalon@mail.mcgill.ca (A.A.); 3 Centre de Recherche de l’ Hôpital Maisonneuve-Rosemont, Centre Universitaire Affilié à l’Université de Montréal, Montreal, QC H1T 3W5, Canada; mohamed.echbicheb@umontreal.ca (M.E.); shigufa.kahn.ali.cemtl@ssss.gouv.qc.ca (S.K.A.); 4 Faculty of Medicine, University of Montreal, Montréal, QC H3T 1J4, Canada; 5 Department of Ophthalmology, Centre Universitaire d’Ophtalmologie (CUO), Hôpital Maisonneuve-Rosemont, University of Montreal, Montréal, QC H1T 3W5, Canada

**Keywords:** retinitis pigmentosa, gene therapy, neuroprotection, stem cell therapy, gene delivery

## Abstract

Retinitis pigmentosa (RP) is the most common inherited retinal dystrophy and remains largely untreatable. This review summarizes RP pathogenesis and critically appraises emerging therapeutic strategies, including gene augmentation, gene editing, RNA-based therapies, gene-agnostic and neuroprotective approaches, stem-cell therapy and their delivery platforms. We compare these strategies according to mutation mechanism, disease stage, durability, delivery route, and current level of clinical evidence. Although several approaches have shown improved structural and functional outcomes in preclinical and early-phase studies, *RPE65*-directed gene augmentation (voretigen neparvovec) remains the only approved therapy, and even it does not halt degeneration. For most modalities, long-term efficacy, safety, durability and clinical validation are not yet established, and translation is further constrained by delivery, immunogenicity, manufacturing, and cost. Rather than a realized paradigm shift, current evidence points to a rapidly advancing but still largely investigational field with successful clinical translation remaining elusive.

## 1. Introduction

Retinitis pigmentosa (RP) is the most common inherited retinal dystrophy (IRD) and affects over 1.5 million individuals worldwide [1,2]. It is classified as syndromic or non-syndromic RP, with the latter restricted to the eye and accounting for 70% to 80% of all cases [2,3]. In most patients, disease progression initiates with rod photoreceptor degeneration, causing peripheral and night vision loss, followed by cone degeneration, which leads to central vision impairment and eventual legal blindness in advanced stages [4]. Most cases follow autosomal recessive (arRP), autosomal dominant (adRP), X-linked (XLRP), and, rarely, mitochondrial inheritance patterns [4]. On multimodal imaging, the hallmark phenotype includes bone-spicule pigment deposition, vascular attenuation, and a waxy pale optic disc, while electroretinography often reveals markedly reduced rod responses with relatively preserved but declining cone function [5,6].

The prognosis in RP is highly variable and depends on the underlying genetic mutation, age of onset, and rate of disease progression. Current management remains largely supportive, aiming to preserve residual vision and optimize functional abilities through low-vision aids, visual rehabilitation, and adaptive strategies [2]. These interventions do not modify the disease course or prevent photoreceptor degeneration, which contributes not only to ongoing visual disability but also to a substantial economic burden [7]. Emerging therapies may help reduce this financial burden by mitigating substantial expenditures associated with disability and long-term care [8].

In recent years, interest in developing disease-modifying approaches has increased. Given the heterogeneity of gene mutations involved in non-syndromic RP, a variety of novel interventions have been explored, including gene therapy, gene-agnostic modifiers, RNA-based therapies, cell-based approaches, and neuroprotective strategies [9,10]. This comprehensive literature review outlines the pathogenesis of RP and highlights recent progress in molecular therapeutic approaches for disease management.

## 2. Methods

This narrative review was informed by searches of PubMed, MEDLINE, and ClinicalTrials.gov, focusing on literature published after 2020. Search concepts included “retinitis pigmentosa” combined with terms related to pathogenesis, gene therapy, genome editing, RNA-based therapy, neuroprotection, stem-cell therapy, and retinal drug delivery. We considered English-language original studies, clinical trials, reviews, and relevant preclinical studies, selecting them based on their relevance to RP and emerging molecular and cellular therapies.

## 3. Pathogenesis of Retinitis Pigmentosa

### 3.1. Mutagenesis

Several inherited forms of RP exist, with arRP the most common (50–60%), followed by adRP (30–40%) and XLRP (5–15%) [3]. RP is largely a monogenic disease, with mutations in more than 80 genes implicated in RP pathogenesis (Table 1). These pathogenic genes commonly encode key components of several vital retina-specific molecular pathways, including phototransduction, RNA splicing, the visual cycle, and ciliary structure. Because a comprehensive catalogue is beyond the scope of this review, Table 1 presents a representative selection of RP-associated genes spanning the three principal inheritance patterns and the major affected pathways.

#### 3.1.1. Phototransduction Cascade

**Figure 1:**
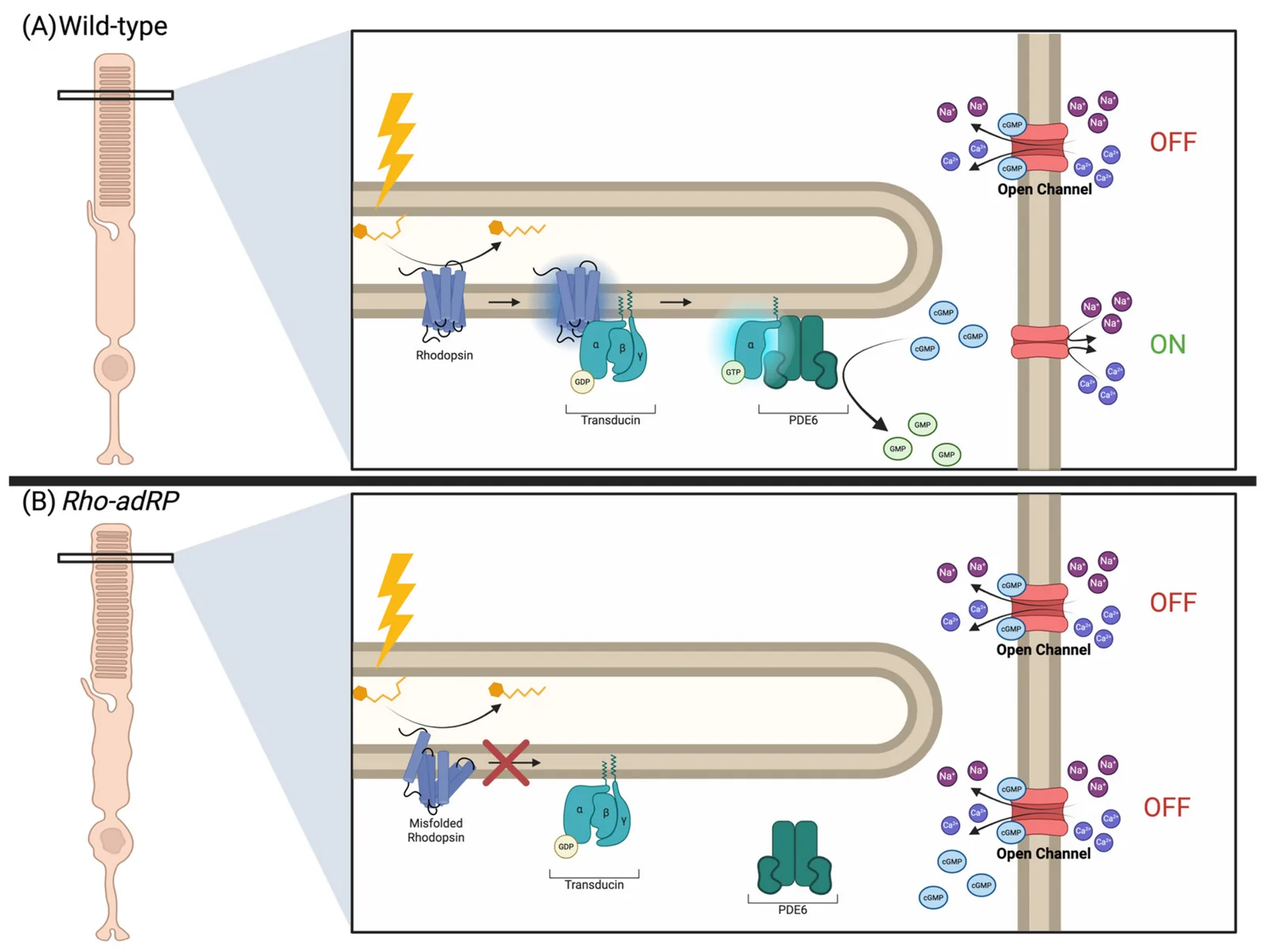
Visual phototransduction cascade. Light is converted into an electrical signal in photoreceptors through a series of reactions known as the phototransduction cascade. (**A**) The disc membranes of the photoreceptors contain visual pigments, including rhodopsin in rods and opsins in cones. The opsins absorb light, causing 11-cis-retinal to isomerize to all-trans-retinal and changing the structure of the opsin. This phenomenon activates the G protein transducin and leads to hydrolysis of cyclic GMP (cGMP) to GMP by phosphodiesterase (PDE). The reduction in cGMP levels causes the CNG channels to close, causing the photoreceptor cell to hyperpolarize and reducing glutamate release. (**B**) adRP-associated rhodopsin mutations impair the ability of rhodopsin to activate the phototransduction pathway. As a result, neither transducin nor PDE is activated. Thus, cGMP levels remain high, the CNG channels remain open, and photoreceptors remain depolarized. Created in BioRender. Tuli, N. (2025) https://BioRender.com/bydhglb.

Mutations affecting components of the phototransduction cascade commonly impair photoreceptor function and drive RP. Most notably, *RHO*, which accounts for approximately 25% of adRP cases, encodes a G protein-coupled receptor (GPCR), rhodopsin, a visual pigment. Rhodopsin localizes within the disc membranes of photoreceptors and functions as a light-sensitive protein that activates phototransduction in dim light (Figure 1) [36]. Over 150 mutations affecting *RHO* have been identified that cause rhodopsin misfolding, defective trafficking and mislocalization, constitutive light-independent activation, altered post-translational modification, and toxic gain-of-function effects [12,36]. Ultimately, these mutations disrupt phototransduction and photoreceptor homeostasis, leading to photoreceptor death [12,36]. Downstream of rhodopsin, *PDE6* mutations, which encode the photoreceptor phosphodiesterase 6 (PDE6) complex, also disrupt phototransduction. PDE6 is a phosphodiesterase composed of a PDE6A/PDE6B heterodimer in rods and PDE6C homodimers in cones, and it hydrolyzes secondary messengers in phototransduction [37]. Mutations affecting these catalytic subunits can cause RP by disrupting rhodopsin signalling (Figure 1) [38].

#### 3.1.2. Spliceosome

**Figure 2:**
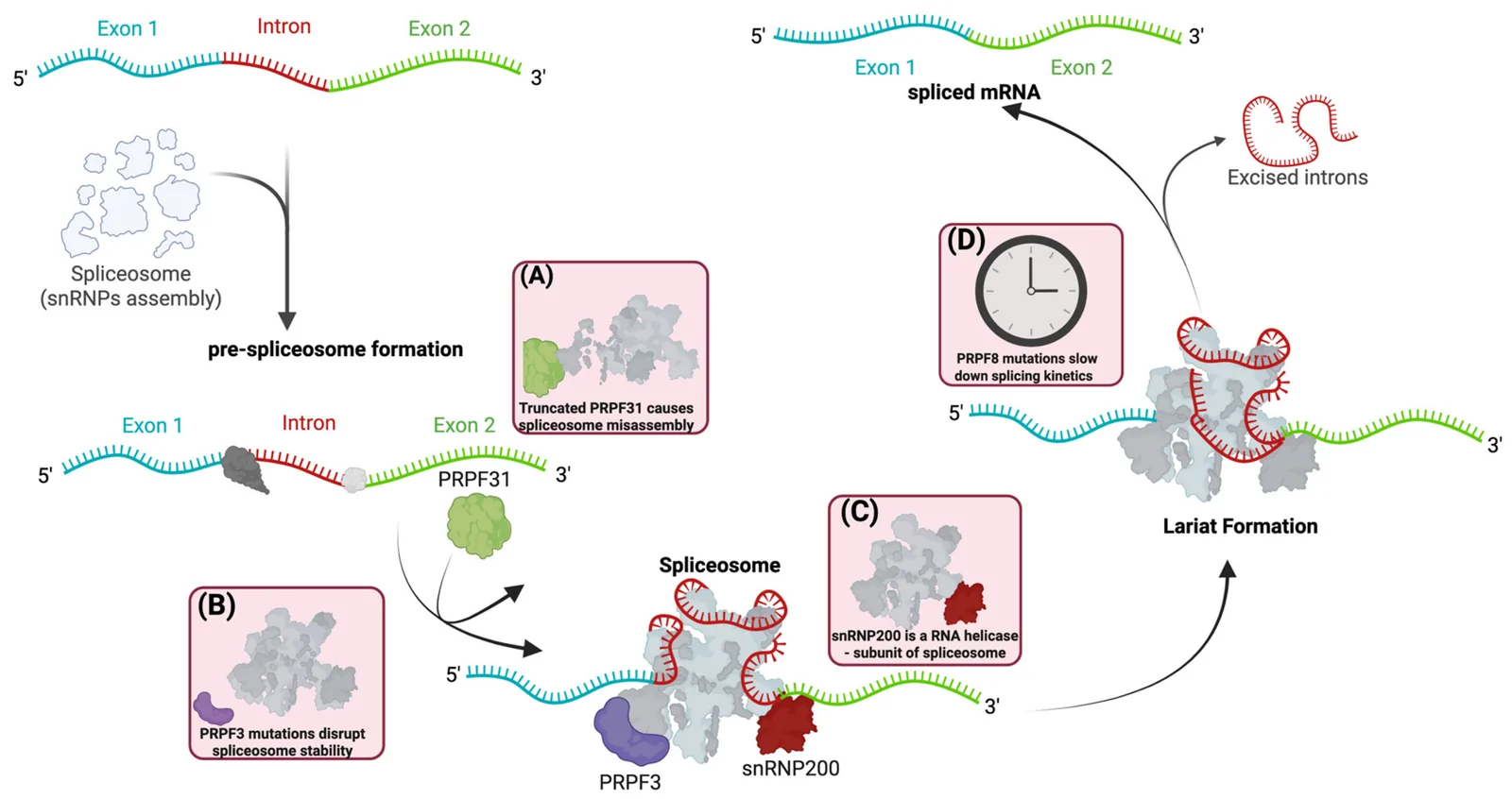
Retinitis pigmentosa-associated mutations affecting the splicing cycle. The splicing cycle consists of snRNPs that assemble to form the pre-spliceosome and then the spliceosome. Once formed, the spliceosome facilitates lariat formation, in which the intron forms a looped intermediate at the branch point. The flanking exons are subsequently joined to form the mature mRNA, while the intron lariat is released. (**A**) PRPF31 mutations cause a truncated protein, leading to misassembly of the spliceosome. (**B**) PRPF3 is integral to spliceosome stability, and adRP-associated mutations disrupt it, thereby impairing spliceosome function. (**C**) snRNP200 is a subunit of the spliceosome and is an RNA helicase required for normal spliceosome function. (**D**) PRPF8 is required for normal kinetics. adRP-associated PRPF8 mutations slow spliceosome kinetics. Created in BioRender. Tuli, N. (2025) https://BioRender.com/n5mipyu.

Another pathway that is often disrupted in RP is the spliceosome. Several components of the U4/U6.U5 tri-snRNP, a major building block of the spliceosome, are associated with RP. These include *PRPF3*, which encodes pre-mRNA processing factor 3 (PRPF3), a protein that disrupts the protein–protein interface required for spliceosome stability [39]. Additionally, PRPF8 interacts with the Brr2 helicase at the catalytic core and, when mutated, disrupts splicing kinetics [40]. Mutations in PRPF31 cause spliceosome misassembly and delay activation [41]. The small nuclear ribonucleoprotein U5 subunit 200 (snRNP200) is also implicated in RP, as *snRNP200* mutations disrupt spliceosome function by impairing U4/U6 unwinding [42] (Figure 2) [43,44]. Interestingly, many of these genes are ubiquitously expressed throughout the body, and tri-snRNP is assembled and functions in all cell types [40,41,45]. However, for unknown reasons, these mutations manifest only in the retina [40,41,45].

#### 3.1.3. Visual Cycle

**Figure 3:**
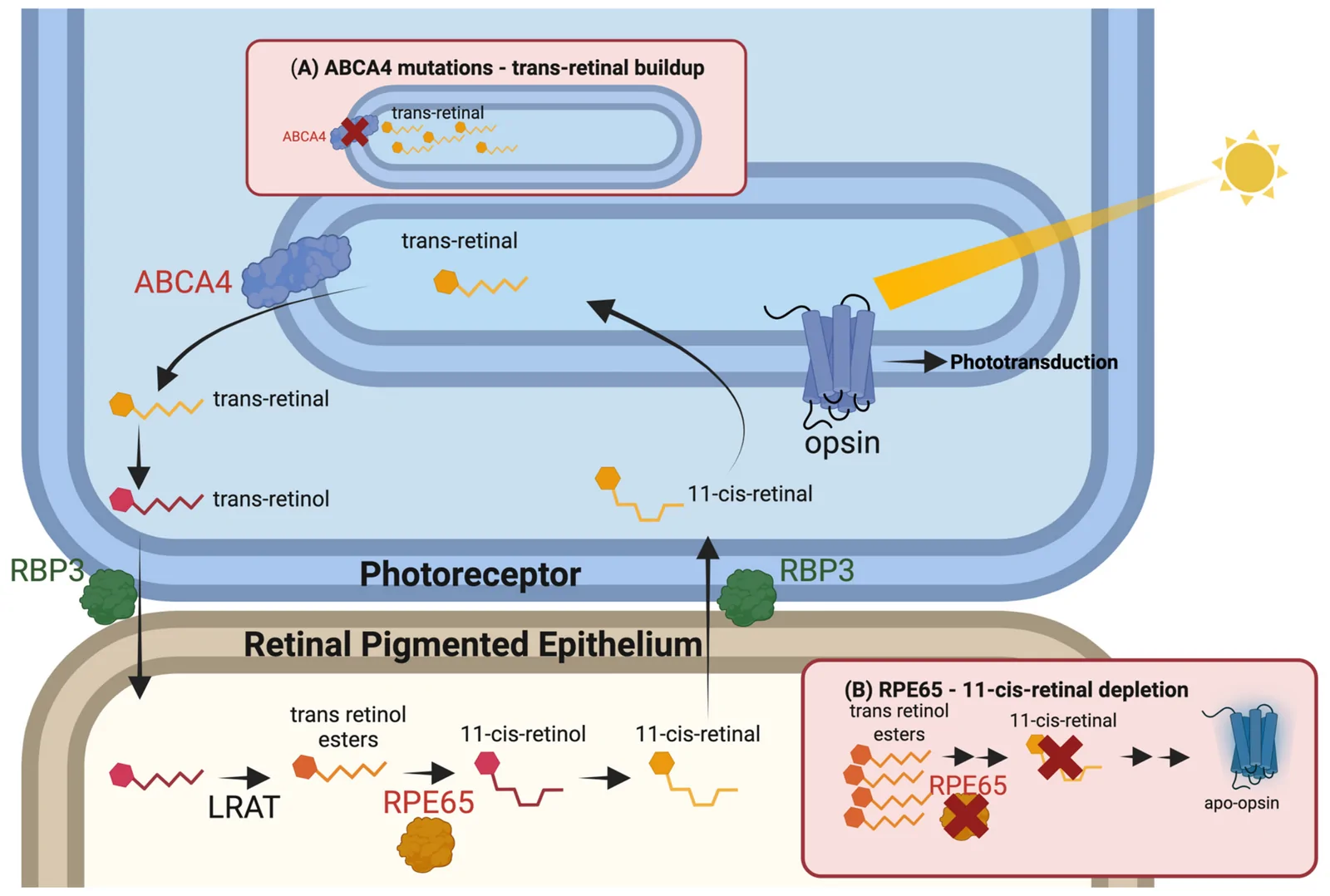
Representation of the visual cycle. The visual cycle encompasses retinal metabolism in the retina, which is involved in phototransduction. During light stimulation of photoreceptors, opsins, such as rhodopsin (known to cause adRP), bind 11-cis retinal, which undergoes photoisomerization to all-trans retinal following photon absorption. All-trans retinal is then transported out of the outer segment disks, facilitated by ABCA4 (known to cause arRP). In photoreceptor cytoplasm, all-trans retinal is converted to all-trans-retinol and transported to the RPE by interphotoreceptor retinoid-binding protein (RBP3). In the RPE, LRAT converts all-trans-retinol to all-trans-retinol esters, which RPE65 then converts to 11-cis-retinol. In the RPE, 11-cis-retinol is converted to 11-cis-retinal, which RBP3 transports back to photoreceptors, restarting the visual cycle. (**A**) With an ABCA4 mutation, all-trans retinal accumulates in the photoreceptor outer segment, disrupting normal metabolism and inducing photoreceptor cell death. (**B**) RPE65 mutations block conversion of all-trans-retinyl esters to 1—cis-retinol, impairing 1—cis-retinal regeneration. This depletes the opsin chromophore and causes all-trans-retinyl esters to accumulate in the RPE, leaving opsin as apo-opsin, which drives photoreceptor dysfunction and degeneration. Created in BioRender. Tuli, N. (2026) https://BioRender.com/h6luce0.

Disruption of the visual cycle can also drive RP. The visual cycle regenerates 11-cis retinal, the chromophore opsins require for phototransduction, through a multicellular pathway involving both the retinal pigment epithelium (RPE) and photoreceptors (Figure 3) [46]. Several forms of RP are attributed to visual cycle mutations, which cause degeneration through two mechanisms [47,48]. Mutations in *ABCA4*, which encodes ATP-binding cassette subfamily A (ABCA4), a transmembrane flippase, lead to the defective clearance of all-trans-retinal from the outer segment of discs [49]. The resulting retinoid adducts condense into cytotoxic products, such as lipofuscin, in the RPE, thereby driving RPE dysfunction and subsequent photoreceptor loss [49]. Alternatively, RPE65 mutations—which convert all-trans-retinol ester to 11-cis-retinol—impair 11-cis-retinal generation [50]. This blocks chromophore supply to opsins, causing all-trans-retinyl esters to accumulate and deposit in the RPE [50]. The resulting deficiency of 11-cis-retinal leaves opsin largely as unliganded apo-opsin, which disrupts normal phototransduction and produces low-level constitutive signalling; together with impaired photoreceptor nutrition and chromophore starvation, this drives progressive rod dysfunction and degeneration [50]. Notably, both *ABCA4* and *RPE65* variants also cause related IRDs, notably Stargardt disease and Leber’s congenital amaurosis (LCA), respectively [29].

#### 3.1.4. Ciliary Structure

Mutations in genes involved in ciliary structure and transport also drive RP, highlighting the importance of outer-segment protein transportation. Retinitis pigmentosa GTPase regulator (RPGR), a connecting-cilium protein localizing to the outer segment of rod photoreceptors, is the most common cause of XLRP, accounting for 70–90% of cases [34]. *USH2A* is the most frequently mutated protein in arRP [51]. It encodes usherin, which localizes to the periciliary region around the connecting cilium and stabilizes the photoreceptor cilium interface [51]. *USH2A* mutations disrupt this interface, leading to progressive photoreceptor degeneration [51]. Notably, usherin also plays a critical role in cochlear hair cells and is associated with a syndromic form of RP, Usher syndrome [47].

### 3.2. Oxidative Stress

Photoreceptors have high metabolic demands, resulting in continuous reactive oxygen species (ROS) generation primarily during mitochondrial oxidative phosphorylation and ATP synthesis. NADPH oxidases (NOXs) also generate ROS, which contribute to immune defences and redox signalling [52]. Elevated ROS levels activate microglia, promoting the release of inflammatory mediators, further amplifying oxidative stress responses [53]. Under homeostatic circumstances, antioxidant defence systems limit ROS-induced damage. These include superoxide dismutase (SOD), which converts superoxide into oxygen and hydrogen peroxide, and glutathione peroxidase (GPx), which reduces hydrogen peroxide to water, helping limit ROS-induced damage [54]. When these protective mechanisms are overwhelmed in RP, oxidative stress damages biological macromolecules, impairs cellular structure and function, and ultimately leads to cell death [53].

Photoreceptor degeneration in RP results from dysfunction in numerous pathways that lead to oxidative stress [55]. The causative mutation drives rod degeneration first, and since rods are the retina’s principal oxygen consumers, their loss causes a relative hyperoxia in the outer retina [56]. This increases ROS generation and creates an oxidative environment that promotes progressive, mutation-independent cone degeneration [56]. This pathology correlates clinically, as peripheral and night vision effects are lost first, followed by central vision in more advanced stages.

Experimental models have demonstrated the influence of ROS in RP. In mice harbouring RP-driving mutations in genes such as *pde6b*, retinal tissues exhibit markedly increased ROS levels compared with wild-type controls [57,58]. Histological analyses show a degenerated retina with evidence of oxidized lipids, proteins, and nucleic acids that accumulate predominantly in photoreceptors [57,59]. These degenerated retinas also contain significant infiltration by microglia and macrophages that express oxidative markers, suggesting these inflammatory cells contribute to the oxidative environment [53,60]. Studies also showed that antioxidant therapy can attenuate degenerative processes. Komeima et al. demonstrated that treatment with vitamin E, a SOD mimetic, vitamin C, and α-lipoic acid significantly reduced oxidized lipid levels and protected against photoreceptor degeneration in mouse models of RP [61]. Additionally, *Nxnl1*-knockout mouse models, in which *Nxnl1* encodes the antioxidant rod-derived viability factor (RdCVF), exhibited an RP phenotype, further highlighting the role of oxidative stress in RP [62].

Substantial clinical evidence reinforces oxidative stress’s role in RP. Indeed, Murakami et al. observed that the vitreous from RP eyes contained fivefold higher levels of 8-oxo-7,8-dihydro-2′-deoxyguanosine (8-oxo-dG), a well-established marker of oxidative DNA damage, than controls [59]. Similarly, Campochiaro et al. found a 2-fold increase in oxidative stress markers in the aqueous humour of RP patients [56], and del Camara et al. reported reduced antioxidant content in aqueous samples from RP patients [63]. Moreover, Vingolo et al. found that peripheral capillary blood from RP patients showed a significant increase in oxygen radicals, and that patients with RP-related cystoid macular edema (CME) had twice as many free radicals [64]. Therefore, it is thought that ROS promote the formation of CME in RP patients through their known role in promoting blood-retina-barrier dysfunction and vascular leakage [64]. Together, these experimental and clinical findings support oxidative stress as a key contributor to RP pathogenesis.

## 4. Gene-Based Therapies

Advances in molecular diagnosis have accelerated the development of genotype-directed therapies for RP. Gene-based treatments can be classified into gene augmentation, suppression, and replacement; genome or RNA editing; and gene-agnostic strategies. Their applicability depends on the underlying disease mechanism, vector capacity, residual retinal structure, and stage of degeneration [48,65].

### 4.1. Gene Augmentation

Gene augmentation delivers a functional wild-type allele without modifying the endogenous locus. It is most appropriate for loss-of-function disorders or complete knockout mutations, where restoring normal gene expression can rescue the retinal phenotype [48,66].

Voretigene neparvovec-rzyl (Luxturna) is an FDA-approved adeno-associated virus (AAV) 2-based gene-augmentation product indicated for patients with confirmed biallelic *RPE65* mutation-associated IRDs [67]. Its approval established the clinical feasibility of subretinal AAV delivery of a functional gene in patients with null or loss-of-function mutations. Luxturna has provided proof of concept for gene therapy in IRDs, driving the next generation of research in this field.

In XLRP animal models and human retinal organoids, subretinal AAV-mediated *RPGR* augmentation preserved photoreceptor structure and function across various stages of RP severity [68,69,70,71]. Several clinical trials have examined both intravitreal and subretinal delivery of an AAV encoding a normal copy of *RPGR* (Table 2). In the open-label phase I/II HORIZON study (NCT03316560), most of the 29 males who received unilateral subretinal AGTC-501 experienced self-limited treatment-emergent adverse events (TEAE) related to the subretinal injection, but the delivery was considered safe and tolerable [72]. Participants treated at the maximum tolerated dose showed the greatest sustained improvement in retinal sensitivity, but researchers abandoned this dose in future studies because these patients developed RPE changes [72]. In a separate randomized, dose-escalation phase I/II study (NCT03252847), 49 participants received subretinal injections containing another AAV encoding wild-type *RPGR* (botaretigene sparoparvovec). As in the previous study, TEAEs were largely related to the surgical procedure [73]. The sponsoring company reported improvements in retinal sensitivity and functional vision up to 52 weeks, prompting a randomized, controlled phase III trial (NCT04671433) that found no improvement in vision in late-stage RP [74].

Gene augmentation is also being investigated for *PDE6A* and *PDE6B* loss-of-function variants. Subretinal AAV-mediated *PDE6B* delivery preserved photoreceptor structure, improved scotopic electroretinogram (ERG) responses, and reduced degeneration in RP rodent models [75,76,77]. This treatment was generally administered early, before retinal degeneration, suggesting a possible preventive role in early RP that has yet to translate clinically. In canine and nonhuman primate studies, delivery of an exogenous *PDE6A*-encoding AAV improved photoreceptor viability [78,79]. These promising results have led to investigation of gene augmentation delivery in the single-arm phase I/II PIGMENT trial (NCT04611503) in patients with biallelic *PDE6A* variants. However, the trial was unsuccessful as subretinal AAV8-*PDE6A* did not produce significant visual improvement at one-year follow-up, and some treated eyes showed central retinal thinning and a decline in visual acuity (VA) [80].

While AAV-mediated gene augmentation is feasible for genes such as *RPGR*, *PDE6A*, and *PDE6B*, the limited packaging capacity of AAV vectors presents a major challenge for larger genes. Biallelic *USH2A* variants cause both Usher syndrome and nonsyndromic arRP. The *USH2A* gene is large and, at 15.6 kb, exceeds the capacity of a single AAV [81]. To address this issue, researchers explored a system using multiple AAV vectors that carry different regions of the gene to increase cargo capacity. However, this approach requires efficient cotransduction and transgene reconstitution, limiting its efficacy [82,83]. A second strategy utilized a nonviral S/MAR episomal vector capable of carrying full-length *USH2A* [81]. This strategy restored *USH2A* expression in patient-derived fibroblasts and partially restored localization of the usherin complex in zebrafish, providing early preclinical proof of concept for an alternative vector to deliver larger genes [81]. Gene augmentation therapies in clinical trials are summarized in Table 2.

**Table 2:** Summary of recently completed and ongoing clinical trials exploring the use of gene augmentation therapy for RP.

Trial ID (Start–End Year)	Trial Phase (Status)	Target Gene	Intervention	Primary Outcome	Published Findings	References
Gene Augmentation						
NCT03252847 (2017–2021)	I/II (Completed)	*RPGR*	Subretinal AAV5-hRKp.RPGR	Incidence of TEAEs	AAV5-hRKp.RPGR had an acceptable safety profile with no reported dose-limiting events	[73]
NCT04794101 (2020–2029)	III (Active)			Incidence of TEAE; improvements in BCVA and low luminance VA (LLVA)	-	-
NCT04671433 (2020–2024)	III (Completed)			Vision-guided mobility assessment	The primary endpoint was not met at 52 weeks. Improvements were observed in LLVA and retinal sensitivity	[84]
NCT06646289 (2024–2030)	II (Active)			Incidence of TEAE; improvements in BCVA and LLVA	-	-
NCT03316560 (2018–2025)	I/II (Active)	*RPGR*	Subretinal rAAV2tYF-GRK1-hRPGRco (AGTC-501)	Incidence of TEAEs	Treatment was well-tolerated with all patients experiencing at least 1 ETAE with complete resolution	[72]
NCT04850118 (2024–2029)	II/III (Active)			Improvements in LLVA	-	-
NCT07174726 (2025–2030)	II (Active)			Changes in macular microperimetry	-	-
NCT06333249 (2021–2027)	II (Active)			Changes in macular microperimetry	-	-
NCT06275620 (2023–2029)	II (Active)			Incidence of TEAEs	-	-
NCT06492850 (2024–2026)	I/II (Unknown)	*RPGR*	Subretinal FT-002	Evaluate safety and tolerance	FT-002 is safe in pediatrics participants, with no serious TEAEs	[85]
NCT05874310 (2023–2027)	I (Recruiting)			Incidence of TEAEs	-	-
NCT04517149 (2020–2029)	I/II (Active)	*RPGR*	Intravitreal AAV-RPGR (4D-125)	Incidence of TEAEs	Well tolerated in the early cohort and serious adverse events reported (published by study sponsor)	[86]
NCT03602820 (2015–2030)	Observational (Active)	*RPE65*	Subretinal AAV2-hRPE65v2 (voretigene neparvovec)	Mobility testing	-	-
NCT03597399 (2019–2025)	Observational (Completed)			Incidence of TEAEs	95% of patients had TEAEs which were mostly mild and procedure related	[87]
NCT00999609 (2012–2030)	III (Active)			Multi-luminance Mobility Testing (MLMT)	AAV2-hRPE65v2 improved visual function with an acceptable safety profile.	[88]
NCT03328130 (2017–2025)	I/II (Terminated)	*PDE6B*	Subretinal AAV5-hPDE6B (HORA-PDE6B)	Incidence of TEAEs	Results Unpublished	-
NCT04611503 (2019–2027)	I/II (Active)	*PDE6A*	Subretinal rAAV.hPDE6A	Incidence of TEAEs	-	-
NCT03374657 (2018–2026)	I/II (Completed)	*RLBP1*	Subretinal AAV8-RLBP1	Incidence of TEAEs and dark adaptation kinetics	Treatment has shown preliminary safety and improvements in dark adaptation kinetics	[89]
NCT01505062 (2012–2019)	I/II (Terminated)	*MYO7A*	Subretinal EIAVMYO7A	Incidence of TEAEs	Two serious TEAEs were reported and the study was terminated due to poor	[90]
NCT06591793 (2024–2029)	I/II (Recruiting)	*MYO7A*	Subretinal AAVB-081	Incidence of TEAEs	Treatment resulted in a favorable safety profile with no serious TEAEs	[91]
NCT03584165 (2018–2026)	III (Completed)	*RPGR*	Subretinal AAV8-RPGR (BIIB112) and choroideremia therapy	Incidence of TEAEs	Results Unpublished	-
NCT06291935 (2023–2026)	I (Recruiting)	*CNGA1*	Intravitreal AAV2.NN-CNGA1 (VG901)	Incidence of TEAEs	-	-
Gene-agnostic modifiers						
NCT05748873 (2023–2030)	I/II (Active)	*RHO*/*PDE6A*/*PDE6B*	Subretinal AAV-RdCVF (SPVNO6)	Incidence of TEAEs	Treatment resulted in favorable safety profile without significant immunogenicity	[92]
NCT05203939 (2023–2027)	I/II (Active)	*NR2E3*	Subretinal AAV5-h*NR2E3* (OCU400	Incidence of TEAEs	The treatment is safe at low and medium doses with possible efficacy	[93]
NCT06574997/NCT06388200 (2024–2027)	Expanded access (Active)			Luminance dependence functional vision	-	-
NCT06388200 (2024–2027)	III (Active)			Luminance dependence functional vision	-	-

### 4.2. Suppression and Replacement

Dominant RP may result from toxic gain-of-function or dominant-negative variants for which adding a normal gene copy alone is insufficient. In these cases, gene augmentation is not a viable strategy, as the exogenous replacement gene will be overcome by the dominant-negative allele [94]. Thus, for toxic gain-of-function mutations, suppression-and-replacement strategies are being pursued that reduce or knock out the mutant alleles while concurrently delivering a replacement construct to restore endogenous expression. Allele-specific suppression preserves the normal allele but is limited to selected variants, whereas mutation-independent suppression may cover a broader range of variants but requires careful regulation of replacement-gene expression and may increase vector burden [48,95,96,97,98].

*RHO* is a major cause of adRP, but pathogenic variants act through diverse mechanisms, including protein misfolding, defective trafficking, altered signalling, toxic gain-of-function, and dominant-negative effects [12]. Allele-specific clustered regularly interspaced short palindromic repeats (CRISPR)/Cas9 suppression has selectively disrupted the mutant *RHO* transgene in a *P23H* rat model, preserving retinal structure and function during long-term follow-up [98]. Another study demonstrated that RNA-specific Cas13X could knock down *RHO-P23H* mRNA transcripts in mice, and replacing them with wild-type transcripts restored *RHO* expression and alleviated RP progression [97]. This RNA-knockdown strategy does not cause genomic alterations; therefore, suppressing the mutant RHO allele would require long-term Cas13X expression or repeated delivery. These two studies were variant-specific, limiting their generalized use in all patients with *RHO* mutations. Two early-phase trials (NCT05805007, NCT06952842) are evaluating subretinal ZVS203e, an rAAV8-delivered CRISPR/Cas9 system designed to disrupt mutant *RHO* expression, with no clinical safety or efficacy reported yet (Table 3).

To address broader allelic diversity, mutation-independent suppression-and-replacement approaches combine disruption of endogenous *RHO* with delivery of a suppression-resistant wild-type construct by targeting a shared sequence in *RHO* that is present regardless of pathogenic variants [95,96]. Cas12i- and Cas9-based strategies delayed degeneration or preserved retinal structure and function by ablating both mutant and wild-type *RHO* expression in mouse models and then replacing it with exogenous AAV-encoded *RHO* modified at the sgRNA target site [95,96]. Two studies demonstrated that this strategy could effectively dampen retinal degeneration in mouse models [68,69]. These studies reported AAV cotransduction rates of 80% and 30–50% for the CRISPR-Cas and replacement-gene vectors, respectively, highlighting the need for co-transduction optimization for CRISPR-based therapies [68,69].

### 4.3. Genome and RNA Editing

**Figure 4:**
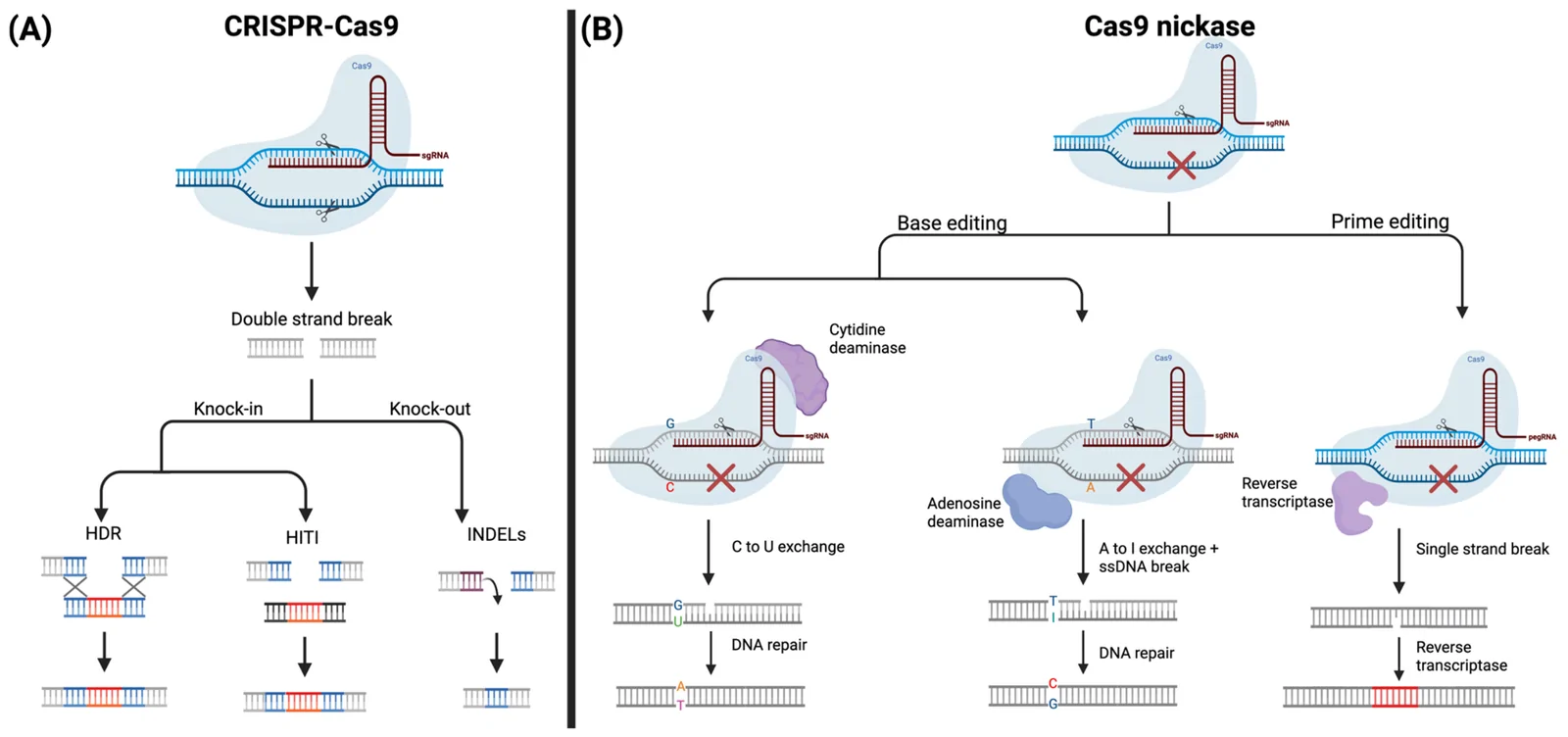
Overview of the mechanism of action of the CRISPR-Cas9 technology. CRISPR-Cas9 includes the Cas9 nuclease, which has 2 catalytic sites and is guided to specific DNA sequences by a guide RNA. (**A**) Traditional CRISPR induces double-stranded breaks that can be used to insert a sequence of interest via homology-directed repair (HDR), in which the sequence is integrated into the genome at a specific site because the flanking sequences are homologous to the host DNA cleaved by CRISPR. Homology-independent targeted integration cuts both the host DNA and a template, which are then joined through NHEJ. CRISPR can also cause knockouts by creating a double-strand break that cellular DNA repair mechanisms repair, introducing insertions or deletions (INDELs). (**B**) Cas9 can be engineered into a nickase, which makes single-stranded breaks. Subsequently, this construct can be fused to base editors (either cytosine or adenosine deaminase), which deaminate their respective nucleotides, causing a single-point DNA alteration. Cytosine deaminase produces a C-to-T change, whereas adenosine deaminase produces an A-to-G change. Additionally, prime editors are CRISPR nickases fused to a reverse transcriptase. They also include a special guide RNA that allows insertion of a short sequence at the single-strand cut site. Created in BioRender. Tuli, N. (2025) https://BioRender.com/npi7kd4.

Genome editing remains largely preclinical and aims to directly alter pathogenic DNA to correct specific variants, largely using CRISPR-Cas9 strategies. Traditional CRISPR-Cas9 constructs target specific sequences to generate double-strand breaks, which are then repaired by nonhomologous end joining (NHEJ), resulting in small insertions and deletions (Figure 4A). Subretinal dual-AAV CRISPR/Cas9 delivery has restored the *RPGR* ORF15 region reading frame in *RPGR* mouse models [99]. However, NHEJ-mediated correction can randomly introduce deleterious insertions and deletions; thus, *RPGR* restoration was infrequent, at approximately 3.3–10.7% of photoreceptors [99]. To overcome this limitation, co-transfection with a repair template can shift cells toward repair of the Cas9-induced break via homology-directed repair (HDR, Figure 4A). A separate study used Cas9-expressing mice and two subretinal AAV vectors carrying the guide RNA and donor template, thereby resolving *RPGR* mutations and producing localized photoreceptor preservation for up to 12 months in mouse models of XLRP [100]. This study demonstrated the technical feasibility in correcting mutations with HDR; however, translation remains limited by heterogeneous repair outcomes and the reduced efficiency of dual-vector cotransduction [101]. Similar CRISPR-Cas9 approaches have been demonstrated in *USH2A* preclinical models, showing that repair via NHEJ and HDR is achievable but not yet optimized for clinical translation [102,103,104].

Researchers have extended CRISPR-based genome-editing tools by fusing Cas9 to deaminases or reverse transcriptase. The resulting complexes are called base editors, which permit selected nucleotide substitutions without double-strand DNA cleavage via deaminases, and prime editors, which can introduce a broader range of substitutions and small insertions or deletions through reverse transcriptase (Figure 4B) [48,66,101,105,106,107]. Researchers have developed an extension of this technology that uses RNA-specific Cas13 fused to adenosine deaminase acting on RNA (ADAR), which modifies mRNA transcripts rather than permanently altering the genome [97,108].

Base editors effectively correct specific point mutations. One study delivered an AAV encoding an adenine base editor that corrected the patient-specific *RHO* c.50C>T (p.Thr17Met) variant in a mouse model, thereby preserving retinal structure and function [109]. Alternatively, RNA-specific base editors restore normal usherin expression in photoreceptors of *USH2A* mouse models [108]. Prime editing can theoretically correct a broader range of substitutions and small insertions or deletions than base editors [110]. Foundational RP studies using prime editors showed they can induce RP-associated mutations in *RPE65* and PRPH2 in animal models [106,107]. More recently, a dual-AAV prime-editing system demonstrated the therapeutic effect of prime editors by correcting the *PDE6B* R560C and Y347X variants in mouse models, resulting in structural and functional rescue [110,111]. Clinical Translation of base and prime editors is limited by mutation specificity, dual-vector cotransduction, and off-target effects [112].

Currently, clinical investigations of CRISPR-Cas9 therapies in RP are sparse and largely limited to CRISPR knockout of dominant alleles rather than editing (Table 3). However, CRISPR-based gene editing has progressed to clinical investigation in LCA. The open-label, single-arm dose-escalation BRILLIANCE phase I/II study investigated EDIT-101, a CRISPR-Cas9 system designed to correct the *CEP290* IVS26 variant [113]. EDIT-101 was administered to 14 participants, of whom six showed improvement in cone-mediated full-field stimulus testing, and nine improved in at least one prespecified visual outcome [113]. This study established the feasibility of gene editing for specific variants that cause IRDs, including those associated with RP.

### 4.4. Gene-Agnostic Modifiers

Gene-agnostic therapies do not directly correct or replace the causal mutation. Instead, they target parallel or functionally redundant pathways that can be enhanced to compensate for the loss of function caused by the disease-associated mutation [114].

The nuclear hormone receptor (NHR) NR2E3 is a transcription factor that is involved in retinal-cell differentiation and homeostasis. NR2E3 has been investigated as a genetic modifier. Genetic modifiers are variants unrelated to the disease-driving mutation that can alter phenotype severity [20,115]. Mutations in *NR2E3* are implicated in retinal degeneration 7 (rd7) mouse models, highlighting their role in retinal homeostasis. Studies have shown that biallelic *NR2E3* knockout in mouse strains with different genetic backgrounds produces different phenotypes, suggesting that genes in parallel with, or in the same pathway as, *NR2E3* can rescue its function and, therefore, the phenotype [116]. Thus, *NR2E3* was investigated as a rescue factor in RP models caused by other mutations. OCU400, an AAV-encoding *NR2E3,* reduced retinal degeneration in *RHO*- and *PDE6B*-RP models by altering retinal biological networks and transcriptional regulation [20]. These promising results led to a phase I/II single-arm study of OCU400 in patients with *NR2E3-* or *RHO*-mediated RP, which reported no serious adverse events, and (n/N = 16/18) patients had stabilization or improvement in the treated eye [117]. A phase III trial is currently underway investigating OCU400 in RP (NCT06388200).

Another gene-agnostic modifier under investigation is RdCVF, a neurotrophic factor encoded by the *NXNL1* gene that supports cone metabolism and survival following rod loss [90]. A study demonstrated that RdCVF can increase cone survival and function in *RHO* mouse models [118,119]. This led to the development of SPVNO6, an AAV encoding RdCVF and its full-length isoform, thioredoxin (RdCVFL), which was shown to reduce vision loss in *RHO* mice [120,121]. These findings led to the phase I/II PRODYGY trial for rod-cone dystrophy (NCT05748873) [120,121]. Gene-agnostic therapies for RP are summarized in Table 2.

### 4.5. Limitations of Gene Therapy

Gene therapy remains challenging, as reflected by only one approved product to date. Even voretigen neparvovec, the sole approved product, does not halt degeneration, as treated eyes show continued photoreceptor loss and waning efficacy over time, underscoring that augmentation cannot rescue cells already lost [88]. Thus, gene augmentation strategies can increase expression, but any therapeutic effect relies on remaining photoreceptors and would be only viable in earlier stages of RP.

Several factors constrain success beyond cell number. Because efficacy depends on a viable photoreceptor substrate, the treatment window is narrow and later-stage disease responds poorly [122]. Subretinal delivery, the standard route, is surgically invasive and, as seen in some of the aforementioned trials, can itself cause focal retinal thinning and detachment [123]. Additionally, injected therapies must transduce effectively, which is dose-dependent. Higher doses can also provoke dose-limiting gene-therapy uveitis and immune responses to the viral capsid, the Cas protein, or the transgene, which limit efficacy [124]. Furthermore, while several preclinical and clinical studies report increased retinal sensitivity, these may not translate into increased mobility or BCVA. Finally, nuclease, base, and prime editors can cause off-target edits at unintended loci as well as unintended on-target outcomes [125]. These include permanent large deletions and rearrangements, and any deleterious edit is irreversible and can worsen visual function [125]. Gene-editing evidence remains almost entirely preclinical, and these limitations must be addressed before effective clinical translation.

## 5. RNA-Based Therapies

In addition to gene therapies, RNA-based therapies encompass several emerging therapeutic strategies for IRDs. These include RNA interference (RNAi), which includes several strategies that alter or suppress RNA translation [10]. This is achieved through exogenous RNAs, including small hairpin (shRNA) and small interfering RNA (siRNA), as well as antisense oligonucleotides (ASOs), which are designed to target specific pathogenic mRNA [10]. Furthermore, endogenous RNAs, such as microRNAs (miRNAs), a subgroup of small noncoding RNAs (ncRNAs), are integral to multiple pathways in the eye and can be manipulated to regulate them concurrently. This section reviews novel technologies for treating RP across these categories.

### 5.1. Noncoding RNAs

ncRNAs play key functional roles in various biological processes. Through their enzymatic and structural functions, ncRNAs regulate cell proliferation, differentiation, and apoptosis [10]. miRNAs are a subgroup of small ncRNAs encoded in the human genome. They are among the most abundant gene families and are highly conserved across species [126].

**Figure 5:**
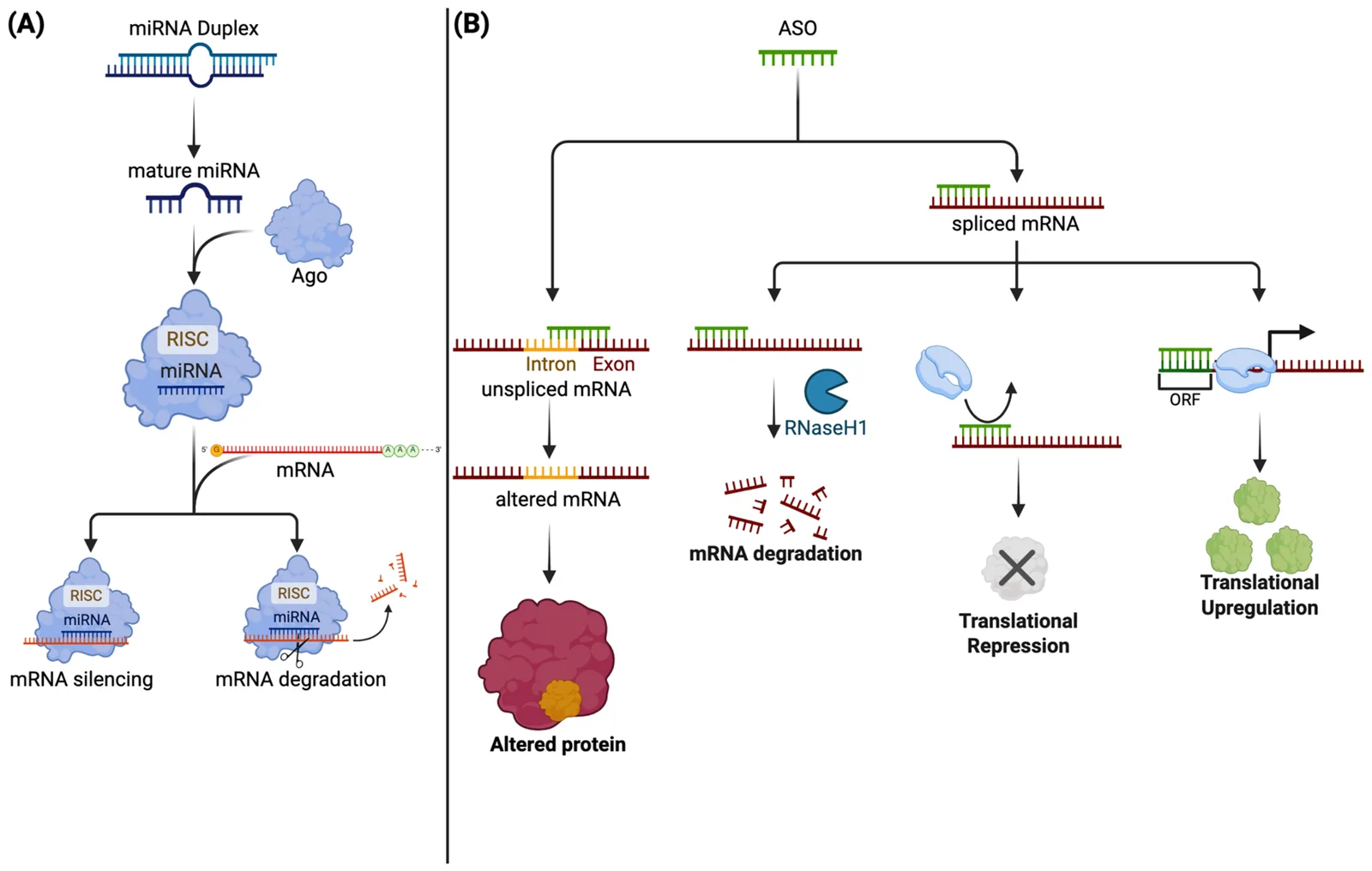
Overview of the molecular mechanisms of RNA-based therapies. Several RNA-based therapies are being investigated for retinitis pigmentosa. (**A**) miRNAs are single-stranded RNAs that are complexed with an Argonaute (Ago) protein to form the RNA-induced silencing complex (RISC) precursor. During this process, one strand of the duplex, known as the guide strand, is selectively retained, while the other strand is discarded. Strand selection is determined by the thermodynamic stability of the duplex end. The strand with the less stable 5′ end is preferentially incorporated into the Ago protein [142,143]. Among the four mammalian Ago (Ago1-Ago4), only Ago2 has been shown to possess endonucleolytic activity capable of inducing mRNA cleavage [144,145]. Thus, the RISC can downregulate gene expression through two primary mechanisms: mRNA degradation and translational repression. The selection between these mechanisms appears to be influenced by the degree of complementarity between the guide RNA and the target mRNA. A higher degree of complementarity favors mRNA degradation, while less complementarity tends to promote translational repression [146]. (**B**) Antisense oligonucleotides are short, single-stranded nucleotide sequences that are designed to target specific RNA. First, they can target unspliced mRNA to correct splicing errors. They can target mature mRNAs and can be designed to cause mRNA degradation, silencing or enhanced translation by targeting upstream open reading frames (ORFs), which usually repress translation. Created in BioRender. Tuli, N. (2025) https://BioRender.com/xgl57w7.

miRNAs are short regulatory RNAs, approximately 22–25 nucleotides long, that are complementary to specific sequences in mRNA. They form RNA-induced silencing complexes (RISCs) when loaded onto Argonaute proteins. miRNAs guide RISCs to complementary mRNAs, which are translationally repressed or degraded (Figure 5A) [127,128]. miRNAs usually target multiple pathways simultaneously, as they recognize complementary sequences shared by multiple mRNAs [128]. Numerous studies have identified many miRNAs enriched in ocular tissues. miRNA microarray profiling in mice has identified at least 78 miRNAs present in the eye, with the miR-183/96/182 cluster strongly expressed in photoreceptors [129]. Similarly, Kapsimali et al. showed that the miR-183/96/182 cluster is also expressed in retinal cell layers [130]. This cluster has been shown to rapidly regulate the voltage-dependent glutamate transporter solute carrier family 1 member 1 (Slc1a1) in photoreceptor cells, a process dynamically modulated by light intensity [131]. This cluster is integral to normal photoreceptor gene expression and can be targeted to correct underlying genetic defects. This is evident in the downregulation of the miR-183/96/182 cluster in retinal organoids derived from hPSCs, which results in abnormal tissue morphogenesis [132,133,134].

Recent studies have extended these findings by exploring endogenous miRNAs as therapeutic targets. A recent study profiled retinal miRNAs in an RP mouse model and identified dysregulation of miR-142a-5p, miR-223-3p, and miR-653-5p [135]. Knocking down miR-142a-5p, a known promoter of inflammation and apoptosis in photoreceptors, using a specific miR-142a-5p decoy delivered via AAV improved retinal function and preserved photoreceptor structure, demonstrating that specific ncRNAs directly contribute to disease progression and can be modulated for therapeutic benefit [135]. Similarly, genetically downregulating miR-181a/b in a murine RP model reduced RPE aerobic glycolysis and slowed photoreceptor degeneration, further establishing the therapeutic potential of endogenous miRNA manipulation for RP [136]. Although no therapeutic strategies have been translated into the clinic, an observational clinical trial is investigating serum miRNAs in *USH2A* patients to explore their potential as biomarkers or disease drivers (NCT06546253).

### 5.2. RNA Interference

ShRNA and siRNA are sequence-specific, exogenously delivered molecules that suppress mRNA through RNAi. They can be applied similarly to the aforementioned gene therapies, with the goal of silencing and subsequently replacing mutant alleles. Mao et al. demonstrated that co-delivery of wild-type *RHO* and siRNA targeting mutant *RHO* mRNA in mice with *RHO*-driven RP effectively slowed degeneration of the photoreceptor and ganglion cell layers, as assessed by ERG [137,138]. Similarly, in a naturally occurring canine model of adRP, AAV-mediated co-delivery of human *RHO* and shRNA targeting canine *RHO* completely preserved photoreceptor structure, as confirmed by histopathological analysis. Long-term follow-up using retinal imaging and ERG further demonstrated sustained functional preservation [139]. The downregulation of valosin-containing protein (VCP), a known pathological driver of RP, using siRNA delivered in magnetic nanoparticles also slowed photoreceptor degeneration in *RHO* P23H rats [140]. These studies collectively highlight that siRNAs or shRNAs targeting specific pathological mRNA transcripts, when delivered with gene-replacement AAVs, can effectively slow photoreceptor degeneration in preclinical models.

### 5.3. Antisense Oligonucleotides

Antisense oligonucleotides (ASOs) are single-stranded RNA or DNA molecules, typically 8–50 nucleotides in length, designed to target specific mRNA sequences. By binding to their target mRNA, ASOs can alter translation by interacting with regulatory elements, such as upstream open reading frames. They can also promote mRNA degradation by recruiting ribonuclease H (RNase H) or modulate pre-mRNA splicing to include or exclude specific exons (Figure 5B) [141].

The first preclinical applications of ASOs in IRD management quickly encountered a major barrier: the molecules were inherently unstable and could degrade before reaching their targets. Phosphorothioate backbone modifications increased ASO stability, enhanced RNase H-mediated cleavage of target mRNA, and reduced renal clearance by promoting binding to plasma proteins [147]. These stabilizing modifications have enabled successful testing of ASOs in preclinical models and, more recently, in clinical trials.

ASOs have shown promise in correcting *USH2A* splicing defects. Several studies have shown that specific intronic defects that lead to pseudexon inclusion and pathogenic *USH2A* mRNA can be corrected with specific ASOs in cell and mouse models of RP [148,149]. Beyond intronic mutations, ASOs can correct *USH2A* variants that disrupt the reading frame by inducing exon skipping in induced PSC (iPSC)-derived photoreceptor precursors from a patient; this effect persisted for up to 6 months, highlighting the translational potential of sustained ASO activity [150]. More recent studies have further expanded exon-skipping approaches, including domain-oriented dual exon skipping in zebrafish RP models and the development of novel ASOs that promote exon 13 skipping and restore photoreceptor ciliary structure in *USH2A-e13* transgenic mice and retinal organoids [151,152]. Collectively, these studies illustrate that ASOs can be strategically tailored to correct both intronic and exonic defects in *USH2A*. Building on these findings, the Stellar trial, a randomized, sham-controlled phase Ib/II clinical trial (NCT03780257) investigated ASO QR-421a in patients with USH2A exon 13 mutations [153]. Initial results shared in conference proceedings showed modest improvements in best-corrected VA (BCVA, approximately 2.2 letters) and increased ellipsoid zone thickness [153]. These results initially led to an open-label extension study and phase II/III clinical trials (NCT05085964, NCT05158296, and NCT05176717), but these were subsequently discontinued for business reasons and efficacy has not been established (Table 4).

Beyond Usher syndrome, QR-1123 is an ASO under investigation as a therapy for adRP caused by *RHO* c.68C>A mutations that selectively targets the mutant mRNA for degradation by RNase H1. Preclinical studies in transgenic mice have shown that QR-1123 can knock down mutant mRNA, thereby slowing the rate of photoreceptor degeneration [10,48,154]. These results have led to the Aurora study (NCT04123626), a phase I/II study examining the safety of QR-1123 in patients with adRP.

ASOs are also being investigated for posterior column ataxia with RP (PCARP), a syndromic form of RP caused by mutations in *FLVCR1* [126]. In a single patient with PCARP secondary to *FLVCR1* deep intronic mutations, a group has begun investigating an FDA-approved mutation-specific ASO in an early-phase I interventional trial (NCT06565572) [155].

Of final note, another splice-modulating ASO, QR-110, is under investigation for *CEP290* splicing-disrupting mutations that cause LCA [156]. Preclinical studies and a single-arm phase I/II clinical trial have established the safety of intravitreal QR-110, with some evidence of improved BCVA and retinal sensitivity [157]. These findings led to phase III trials (NCT06891443, NCT04855045, NCT03913143) investigating the efficacy of QR-110. While many RNA-based therapies have shown promise in preclinical trials, like gene therapies, translating them into clinically meaningful outcomes remains elusive.

**Table 4:** Summary of ASO clinical trials for RP and related IRDs.

Trial ID (Start–End Year)	Trial Phase (Status)	Target Gene	Intervention	Primary Outcome	Published Findings	References
NCT03140969 (2017–2019) NCT03913130 (2019–2022)	Ib/II (Completed)	*CEP290*	Intravitreal QR-110 (Sepofarsen)	Incidence of TEAEs	The treatment had a manageable safety profile and resulted in improved VA	[157]
NCT06891443 (2025–2028)	III (Active)			Change in BCVA	-	-
NCT04855045 (2021–2023)	II/III (Active)			Incidence of TEAEs	-	-
NCT03913143 (2019–2023)	II/III (Active)			Change in BCVA	-	-
NCT05085964 (2021–2022)	II (Terminated)	*USH2A exon 13*	Intravitreal QR-421a (Ultevursen)	Incidence of TEAEs	Results Unpublished	-
NCT05158296 (2021–2022)	II/III (Terminated)			Change in BCVA	Results Unpublished	-
NCT05176717 (2021–2022)	II/III (Terminated)			Change in mean sensitivity on static perimetry	Results Unpublished	-
NCT03780257 (2019–2022)	Ib/II (Completed)			Incidence of TEAEs	QR-421a was safe and tolerable, with results showing stabilization of retinal structure and function	[153]
NCT06627179 (2024–2027)	II (Active)			Change in ellipsoid zone on OCT	-	-
NCT04123626 (2019–2022)	I/II (Active)	*RHO*	Intravitreal QR-1123	Incidence of TEAEs	-	-
NCT06565572 (2023–2028)	I (Enrolling)	*FLVCR1*	Intravitreal nL-FLVC-001	Incidence of TEAEs	-	-

## 6. Neuroprotective Agents

**Figure 6:**
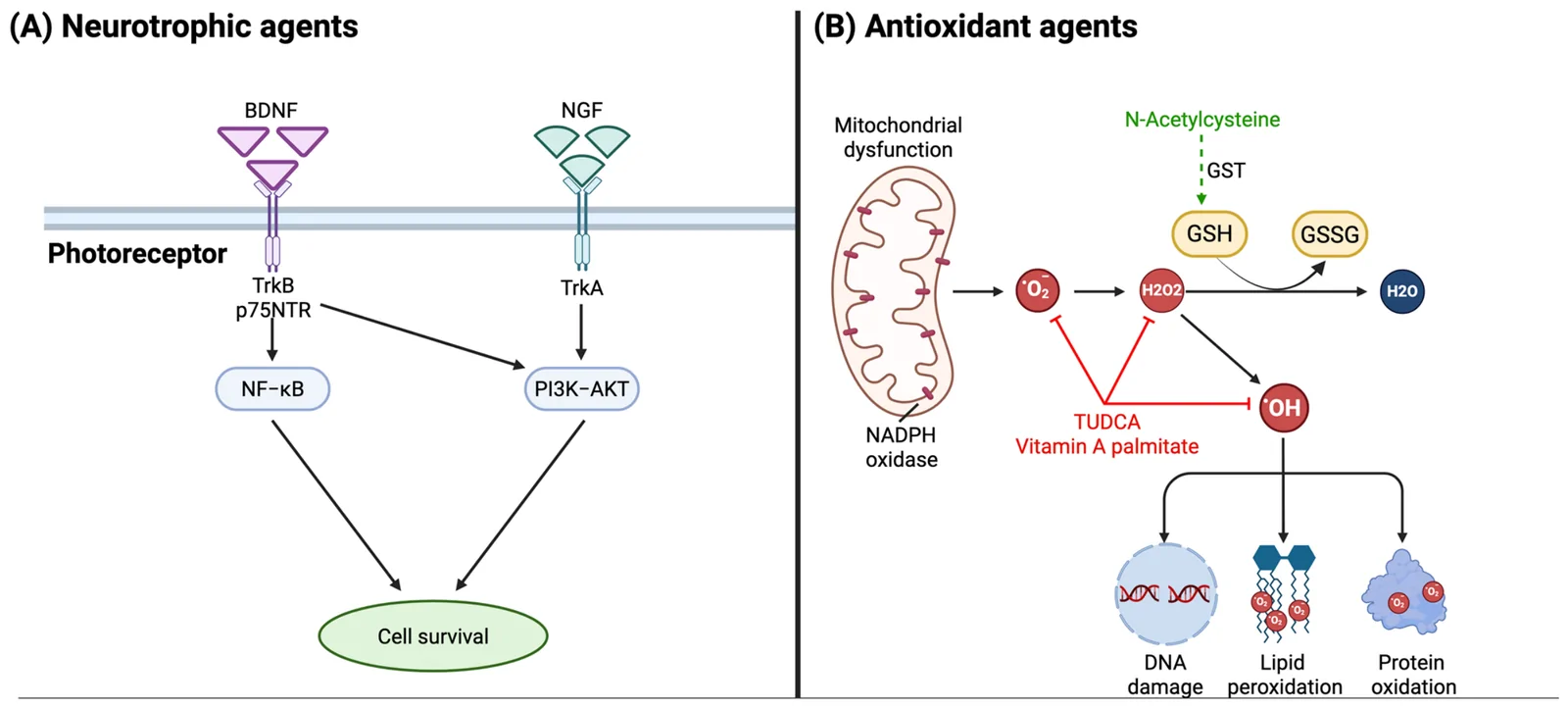
Overview of molecular signalling pathways targeted for the treatment of RP. Several novel therapies are being investigated that target specific metabolic pathways. (**A**) Neurotrophic factors, including BDNF and NGF, all target cell signalling pathways that promote cell survival. (**B**) Reactive oxygen species produced by mitochondrial dysfunction promote cell dysfunction by causing DNA damage, lipid peroxidation or protein peroxidation. The natural antioxidant reduced glutathione (GSH) reduces ROS, producing water and oxidized glutathione (GSSG). N-acetylcysteine is a precursor of GSH and can be used therapeutically to enhance antioxidant defences. TUDCA and vitamin A also act on this pathway by directly eliminating ROS. Created in BioRender. Tuli, N. (2025) https://BioRender.com/nnzyr9p.

Neuroprotective agents, including neurotrophic factors, anti-apoptotic molecules, and antioxidant agents, are therapeutic compounds designed to prevent cellular degeneration in various diseases (Figure 6) [153]. In RP, their main objective is to delay photoreceptor degeneration, reduce oxidative stress, and preserve the function of the remaining retinal cells.

### 6.1. Neurotrophic Factors

Neurotrophic factors play a crucial role in promoting neurite outgrowth and nervous system development [158]. The eye produces various neurotrophic factors, including ciliary neurotrophic factor (CNTF), brain-derived neurotrophic factor (BDNF), and nerve growth factor (NGF) [159].

#### 6.1.1. Ciliary Neurotrophic Factor

Ciliary neurotrophic factor (CNTF) belongs to the interleukin-6 (IL-6) cytokine family and exerts its effects by binding to a heterotrimeric receptor complex consisting of CNTF receptor α (CNTFRα) [160,161,162,163]. CNTFRα is expressed on the membranes of Müller glial cells [164,165] as well as on rod and cone photoreceptors [166,167,168,169,170]. Two sham-controlled clinical trials (NCT00447993, NCT00447980) investigated CNTF implants in patients with early- and late-stage RP, but neither demonstrated a therapeutic benefit [171]. Thus, CNTF was largely abandoned as a therapeutic option for RP [171]. Later animal studies showed that while CNTF slowed photoreceptor death, it caused rods to transdifferentiate into bipolar-like phenotypes that suppressed rhodopsin, thereby reducing function [172,173].

#### 6.1.2. Brain-Derived Neurotrophic Factor

Brain-derived neurotrophic factor (BDNF) is another neurotrophic agent under investigation for RP. Strong preclinical evidence shows it can support photoreceptor survival and occasionally improve ERG responses [174,175,176,177,178,179]. In the retina, BDNF localizes to the plexiform layers and outer nuclear layer, while its receptor TrkB is found in the inner plexiform layer and select photoreceptor outer segments [180,181,182]. BDNF-TrkB binding activates PI3K/Akt, MAPK, and PLCγ signalling, upregulating anti-apoptotic proteins such as Bcl-2 and Bcl-XL while inhibiting pro-apoptotic pathways [174] (Figure 6A).

Initial studies demonstrated that in vivo electroporation of BDNF could promote photoreceptor survival in rats with photoreceptor degeneration [183]. More recently, sustained delivery via gene therapy methods, primarily AAV-mediated BDNF/TrkB delivery, has shown promise in preventing RGC degeneration in glaucoma mouse models [184,185]. Furthermore, combined administration of CNTF and BDNF in RP mouse models reduced photoreceptor apoptosis and further upregulated endogenous CNTF and BDNF, suggesting glial cell-mediated positive feedback [186]. Notably, neither CNTF nor BDNF could rescue photoreceptors alone, and this effect required combined administration [187]. Another study in *RHO* mutant mice supported this synergistic effect, showing that BDNF alone did not protect against retinal degeneration [174,188].

Clinical translation remains limited by BDNF’s short intraocular half-life, the possibility of receptor desensitization or downregulation with repeated exposure, and the challenge of sustained posterior-segment delivery [174]. Currently, sustained delivery would require multiple intravitreal injections (IVIs); however, recent work has explored viral vectors [184,185,189] and cell-based platforms [190] for prolonged BDNF administration. To date, BDNF has not been evaluated clinically in RP and all evidence remains preclinical.

#### 6.1.3. Nerve Growth Factor

Nerve growth factor (NGF) is synthesized locally within the retina by several cell types, including Müller glia, RGCs, and possibly photoreceptors, and exerts its biological effects through the high-affinity tropomyosin receptor kinase A (TrkA) receptor and the low-affinity p75 neurotrophin receptor (p75NTR) [191,192,193]. Activation of TrkA promotes photoreceptor survival through downstream pro-survival signalling pathways, including PI3K/Akt and MAPK/ERK, whereas p75NTR signalling is context-dependent and may promote cell survival or apoptosis depending on receptor interactions and the local microenvironment [191,192,193].

Early observations noted that NGF delays retinal degeneration in inherited RP models [194]. More recently, Rocco et al. evaluated topical NGF administration in RCS rat models of photoreceptor degeneration and demonstrated enhanced photoreceptor survival [192]. Furthermore, experiments using isolated photoreceptors showed that NGF directly promoted photoreceptor survival and neurite outgrowth through TrkA activation, supporting a direct neuroprotective effect rather than one mediated exclusively through surrounding retinal cells [192]. Additional evidence for the therapeutic potential of NGF comes from Jian et al., who demonstrated that bone marrow-derived mesenchymal stem cells promoted endogenous retinal repair in RCS rats through activation of the NGF/TrkA signalling pathway, with blockade of NGF largely abolishing their neuroprotective effects [195].

Clinical applications of NGF are limited, with one pilot study conducted in humans. Falsini et al. found that NGF eye drops were well tolerated in patients with RP, with a minority showing improvements on visual field testing and in ERG responses [196]. The LUMOS study (NCT02110225), a phase I/II clinical trial investigating NGF eye drops in RP, was completed without a published peer-reviewed article or any follow-up clinical trials [197]. Thus, the clinical benefit of NGF remains unproven.

### 6.2. Antioxidants

Oxidative stress is considered a major contributor to secondary cone degeneration in RP. Following rod photoreceptor loss, reduced oxygen consumption increases outer-retinal oxygen tension, leading to excessive generation of reactive oxygen species (ROS) [61]. The resulting oxidative damage contributes to progressive cone dysfunction and apoptosis, providing a mutation-independent rationale for antioxidant therapy to preserve residual cone structure and function [61].

#### 6.2.1. Vitamins

Vitamin A has historically been investigated as a nutritional therapy for RP because of its essential role in the visual cycle. In the landmark randomized trial by Berson et al., vitamin A palmitate (15,000 IU/day) modestly slowed the decline in 30 Hz cone flicker ERG amplitude, whereas high-dose vitamin E (400 IU/day) was associated with a more rapid decline [198]. However, a recent genotype-based reanalysis by Comander et al. failed to confirm a generalized neuroprotective benefit of vitamin A while reaffirming the detrimental effect of vitamin E [199]. Today, published guidelines do not support vitamin A for RP and advise against high-dose vitamin E supplementation [200].

#### 6.2.2. Tauroursodeoxycholic Acid

Tauroursodeoxycholic acid (TUDCA) is a hydrophilic bile acid with anti-apoptotic, anti-inflammatory, and antioxidant properties that targets common downstream pathways involved in photoreceptor degeneration (Figure 6B) [201]. Systemic TUDCA reduced photoreceptor apoptosis and preserved retinal structure and ERG function in mouse models of RP [202,203]. Subsequent studies have further supported these findings across multiple RP models. Drack et al. demonstrated preservation of retinal structure and function in slowly, but not rapidly, progressing mouse models, suggesting a mutation-specific effect [204]. More recently, sustained intravitreal delivery of TUDCA using biodegradable PLGA microspheres preserved retinal architecture, synaptic connectivity, and ERG responses in *RHO*-rat models, highlighting a promising strategy for long-term ocular drug delivery [205]. TUDCA evidence remains preclinical, and promising results potentially warrant clinical translation.

#### 6.2.3. N-Acetylcysteine

N-acetylcysteine (NAC) is an antioxidant that replenishes intracellular glutathione and reduces oxidative stress [206]. In experimental RP models, oral NAC reduced oxidative damage, preserved cone structure and function, and promoted long-term cone survival [207]. Topical NAC also reduced retinal oxidative stress and improved cone survival, supporting the role of oxidative damage in secondary cone degeneration [207]. The findings led to a phase I dose-escalation study, in which oral NAC was generally well tolerated in patients with moderately advanced RP [208]. All treatment cohorts showed improvements in BCVA, while macular sensitivity improved significantly only in the highest-dose cohort; however, the study detected no significant change in ellipsoid-zone width and was not designed to establish efficacy [208]. The ongoing NAC Attack Phase III randomized controlled trial (NCT05537220) is currently evaluating whether long-term oral NAC can safely slow cone degeneration and preserve visual function in patients with RP [209].

#### 6.2.4. Combined Antioxidants

Komeima et al. provided pivotal evidence supporting oxidative stress as a mechanism of secondary cone death in RP [61]. In mouse models of RP, they demonstrated that a combination of α-tocopherol, ascorbic acid, manganese(III) tetrakis(4-benzoic acid) porphyrin (MnTBAP), and α-lipoic acid significantly reduced retinal oxidative damage, promoted cone survival, increased cone opsin expression, and partially preserved photopic ERG responses [61]. Notably, individual antioxidants provided less protection than the combined regimen, suggesting that simultaneously targeting complementary oxidative pathways may offer greater protection than individual components alone [61]. Although these findings strongly support oxidative stress as a therapeutic target, combination antioxidant therapy remains limited to preclinical investigation. Overall, no neuroprotective strategies have demonstrated functional benefits in RP. Table 5 summarizes clinical trials evaluating neuroprotective therapies for RP.

### 6.3. Cell Death Pathways

Despite the remarkable genetic heterogeneity of RP, diverse disease-causing mutations converge on a limited set of regulated pathways of photoreceptor cell death. Although apoptosis was historically considered the predominant mechanism of photoreceptor degeneration, accumulating evidence indicates that multiple regulated pathways contribute to disease progression, including cyclic guanosine monophosphate (cGMP)-dependent protein kinase G (PKG), poly(ADP-ribose) polymerase (PARP), histone deacetylases (HDACs), calpains, and receptor-interacting protein kinase (RIPK)-mediated necroptosis [211,212]. The relative contribution of these pathways likely varies with the underlying genetic mutation, photoreceptor subtype, and disease stage. Importantly, because these pathways lie downstream of diverse genetic defects, they represent attractive mutation-independent therapeutic targets [213].

#### 6.3.1. cGMP-PKG Signalling

Accumulation of intracellular cGMP is considered one of the earliest pathogenic events in several forms of RP, particularly those associated with *PDE6* mutations [212]. Excessive cGMP can promote photoreceptor death through aberrant PKG activation, which has been linked to HDAC, PARP, and calpain activity, and to persistent activation of cyclic nucleotide-gated (CNG) channels, resulting in calcium dysregulation and calpain activation [212]. Both pathways trigger cell death signalling, highlighting the prevalence of caspase-free cell death in RP [213]. Consequently, PKG inhibition has emerged as one of the most promising mutation-independent therapeutic strategies.

Treatment with the novel PKG inhibitor CN238 significantly preserved photoreceptor survival and function in mouse models of RP and also protected against RGC degeneration [214]. These findings were subsequently extended to an adRP model harbouring a *RHO* mutation, in which treatment with the PKG inhibitors CN03 and CN238 reduced photoreceptor death, preserved cone survival, and maintained retinal light responses [215]. Together, these studies position PKG as an upstream therapeutic target capable of modulating several cell-death pathways, though it has yet to be translated clinically.

#### 6.3.2. PARP-Dependent Cell Death

Excessive PARP activation is another hallmark of inherited retinal degeneration. Although PARP normally facilitates DNA repair, sustained activation depletes intracellular NAD^+^ and ATP, leading to a regulated form of cell death known as parthanatos [216]. PARP1-deficient mice exhibited normal retinal architecture and function, while retinal explants lacking PARP1 were markedly more resistant to photoreceptor degeneration induced by pharmacological PDE6 inhibition, supporting a causal role for PARP1 in cGMP-associated photoreceptor death [217].

Subsequent pharmacological studies further validated PARP as a therapeutic target. Treatment with the clinically approved PARP inhibitor olaparib significantly reduced PARP activity, decreased cGMP accumulation, and delayed photoreceptor degeneration in mouse models of RP [218]. Two additional repurposed PARP inhibitors, BMN-673 and 3-aminobenzamide, also reduced rod photoreceptor death in rd1 retinal explants [219]. Furthermore, PARP inhibition suppressed calpain activation, whereas calpain inhibition did not alter PARP activity, indicating that PARP acts upstream of calpain within the same degenerative pathway [220].

Notably, contrasting findings were recently reported in a *RHO* mouse model, in which all PARP inhibitors tested produced marked, dose-dependent rod photoreceptor toxicity rather than neuroprotection [221]. This effect appeared independent of rhodopsin expression and contrasted with the neuroprotective effects consistently observed in recessive *PDE6*-associated models, while cone photoreceptors appeared relatively unaffected [221]. These findings suggest that the therapeutic effects of PARP inhibition may depend on the underlying genetic subtype; therefore, tailoring PARP inhibition to specific genotypes may be required [221]. These contrasting results warrant further clarification before translation into humans.

#### 6.3.3. Calpain-Mediated Photoreceptor Degeneration

Calpains are calcium-dependent cysteine proteases activated following excessive intracellular calcium influx. Persistent cGMP accumulation promotes sustained opening of CNG channels and voltage-gated calcium channels, causing calcium overload and calpain-mediated degeneration [222]. Calpain inhibitors administered to mouse models of RP reduced photoreceptor death [222]. Importantly, these beneficial effects were observed only with selective calpain inhibitors, whereas other non-selective inhibitors were toxic to the retina [222]. Notably, a more recent study showed that PARP and HDAC inhibitors could reduce calpain activity; however, combining the inhibitors did not produce a synergistic effect, suggesting that all three factors operate within overlapping pathways [223]. Together, these findings highlight the importance of regulating calpain in RP, with PARP or HDACs as alternative targets if direct calpain inhibition continues to cause retinal toxicity. Like other cell-death-targeting therapies, these studies remain entirely preclinical, and further clarification is needed before human studies.

#### 6.3.4. HDAC-Mediated Degeneration

Aberrant HDAC activation has emerged as another important contributor to photoreceptor degeneration. Excessive HDAC activity precedes photoreceptor degeneration in mouse models of RP, and inhibiting HDACs significantly attenuates photoreceptor death, identifying HDACs as potential therapeutic targets in RP [224]. Further studies strengthened the therapeutic potential of HDAC inhibition. Treatment with the HDAC inhibitor suberoylanilide hydroxamic acid (SAHA) delayed photoreceptor degeneration while suppressing HDAC and PARP activity, providing additional evidence that these pathways are mechanistically linked [225]. Moreover, a single intravitreal administration of an HDAC inhibitor during advanced disease stages significantly prolonged cone survival and preserved cone-mediated visual function in two RP mouse models [226]. However, another study advised caution after testing several HDAC inhibitors in adRP mouse models, finding that some could induce photoreceptor cell death and proliferation [227]. Indeed, HDAC inhibition with SAHA yielded strong anti-apoptotic effects but also increased non-apoptotic cell death, highlighting potential toxicity [227]. However, other HDAC inhibitors, such as MTP, yielded strong photoreceptor protection, highlighting both the complexity and the potential of targeting HDACs as a therapy for RP [227]. Thus, while certain HDAC inhibitors have shown promise in preclinical studies, others can cause harm, highlighting the fine balance in targeting HDACs as a therapy for RP.

#### 6.3.5. RIPK-Mediated Necroptosis

RIPK is a key player in necroptosis, a form of necrotic cell death [228]. Studies have shown that RIPK3 can drive retinal neuroinflammation and photoreceptor loss in mouse models and human RP samples [228,229,230,231]. Accumulating evidence suggests that secondary cone degeneration, rather than the primary genetically driven rod degeneration, may involve distinct regulated cell-death mechanisms. In line with this observation, genetic deletion of RIPK3 did not prevent rod photoreceptor loss but significantly preserved cone survival, while pharmacological inhibition of RIPK similarly delayed cone degeneration and maintained cone function. These findings indicate that RIPK-mediated necroptosis primarily contributes to secondary cone degeneration rather than initial rod degeneration in preclinical models, suggesting its potential to preserve cone photoreceptors and central vision in advanced RP.

## 7. Stem Cell Therapy

Photoreceptors are post-mitotic retinal neurons with limited regenerative capacity; therefore, their loss in RP is largely irreversible. Stem cell-based therapies pursue two distinct objectives: retinal cell replacement and cell-based neuroprotection. Replacement strategies transplant photoreceptor precursors or organized retinal tissue to restore lost cells and establish connections with the host retina. Neuroprotective approaches instead aim to preserve residual retinal function through trophic, paracrine, immunomodulatory, or vascular effects of cell-based therapies [65,232]. Stem cell-based approaches have shown promise in preclinical models; however, functional cell integration that provides clinical benefit has yet to be achieved.

### 7.1. Retinal Cell Replacement

#### 7.1.1. Stem Cell Sources

Human pluripotent stem cells (hPSCs) include human embryonic stem cells (hESCs) and human induced pluripotent stem cells (hiPSCs). hESCs are generally used as allogeneic products and raise ethical concerns related to embryo derivation, as well as alloimmune risk. hiPSCs are generated by reprogramming somatic cells and may be used as autologous or allogeneic products, avoiding embryo-related ethical concerns [233].

hPSCs can be differentiated into photoreceptor-lineage cells directly or through human PSC-derived retinal organoids [234,235,236,237]. Retinal organoids recapitulate several developmental and structural features of the human retina, but they do not fully reproduce maturation, the vascular environment, synaptic organization, or the physiology of the adult human retina [238]. However, they can generate either dissociated photoreceptor precursors or organized retinal tissue layers, termed retinal sheets [239]. The latter provides better cell organization but requires more complex surgical procedures [239,240,241]. In comparison, subretinal delivery of dissociated photoreceptors is relatively simple, but it is largely limited by poor cell survival and uneven distribution due to gravity [239].

#### 7.1.2. Dissociated Photoreceptor Transplantation

Early preclinical studies have shown that transplanted photoreceptor precursors can survive within the degenerating retina and contribute to visual function. Pearson and colleagues subsequently transplanted postnatal mouse rod photoreceptor precursors into mice with defective phototransduction, showing donor cells within the host outer nuclear layer with apparent photoreceptor morphology and light-responsive synapses [242]. Furthermore, transplantation was associated with improved visually mediated responses in animals [242]. Notably, this model did not have significant outer-retinal cell loss characteristic of advanced RP; therefore, these results can only be extrapolated to early RP.

The precise therapeutic mechanism of transplanted cells has been controversial, with some arguing against integration and synapse formation. Rather, several animal-based studies have demonstrated donor–host cytoplasmic material transfer, as fluorescent proteins and other cytoplasmic constituents have been shown to pass between donor and host photoreceptors [243,244,245,246]. This transfer of cytoplasmic material mimics integration as host cells acquire donor-associated markers [243,244,245,246,247,248]. This transfer occurs largely in early stages of disease, with preserved photoreceptors [243,244,245,246]. In more advanced stages of degeneration, when virtually all photoreceptors are lost, evidence in animal models shows that transplanted photoreceptor progenitors can integrate and form synaptic connections with the host retina [239,249,250,251,252,253,254,255]. Thus, it is postulated that in less advanced disease, where host photoreceptors remain, the transplanted cells’ therapeutic effect is mediated by material exchange, whereas in advanced disease with significant host photoreceptor loss, donor cells integrate.

#### 7.1.3. Retinal Sheet Transplantation

At advanced stages of RP, the outer-retinal architecture is severely depleted, making retinal sheets—which supply an organized photoreceptor layer—a particularly relevant approach. In animal models of advanced RP or retinal degeneration, studies have shown that transplanted retinal sheets can integrate with the host retina, with subsequent improvements in visual function [253,255,256,257,258]. Mandai and colleagues transplanted mouse iPSC-derived retinal sheets into end-stage rd1 mice, which carry a mutation in *PDE6B,* and reported graft maturation, host-graft synaptic contacts, and visually responsive RGCs in a subset of animals [256]. Hirami and colleagues subsequently translated this strategy into humans. They transplanted allogeneic hiPSC-derived retinal organoid sheets into one eye of two patients with advanced RP: one patient harbouring a mutation in the eyes shut homolog (*EYS*) gene associated with arRP, and the other presumed to have adRP but with no identified causative mutation [259]. The graft remained stable for 2 years, with increased retinal thickness and no serious adverse events reported [259]. Exploratory functional assessment showed no improvement in VA or Humphrey visual field testing, whereas full-field stimulus testing suggested improvement in the transplanted eye of the participant whose preoperative VA was hand motions [259]. Notably, compared with the untreated eye, visual function loss was less pronounced in the transplanted eye during follow-up [259]. This study supports the feasibility and preliminary safety of retinal sheet transplantation in RP patients, but larger future studies are needed to establish true clinical efficacy.

Following the initial safety study, two additional phase I/IIa trials are evaluating allogeneic iPSC-derived photoreceptor precursor cells (OpCT-001; NCT06789445) in primary photoreceptor disease and iPSC-derived retinal sheets (DSP-3077; NCT06891885) in RP. Clinical trials of pluripotent stem cell-derived RPE, photoreceptor-precursor, and retinal-sheet therapies are summarized in Table 6.

#### 7.1.4. Remaining Challenges

##### Integration Versus Material Transfer

The exchange of cytoplasmic material between donor and host photoreceptors affects transplantation outcomes [243,244,245,246]. As a result, the method used to identify donor cells is critical to distinguish between donor-cell integration and material transfer [243]. Even nuclear proteins such as Cre recombinase may transfer between donor and host [243]. Therefore, donor identity should be confirmed using complementary methods, including human nuclear antigen, human-specific DNA or RNA in situ hybridization, sex-mismatched Y-chromosome fluorescence in situ hybridization, or direct genotyping [244,267,268].

##### Synaptic Connectivity

There is strong evidence that donor cells can form synapses with the host retina in animal models [239,249,250,251,252,253,254]. Cell integration does not equate to the formation of functional, visually significant synaptic connections. Transplanted photoreceptors must mature, respond to light, and form functional synapses with host retinal neurons to confer any clinical benefit. While preclinical studies provide strong evidence for integration, especially in more advanced degeneration, some studies have failed to demonstrate visual recovery, while others have shown promising improvements in light-evoked behaviour in treated animals [253,254]. Therefore, histological evidence of synaptic-marker expression should be consistently supported by electrophysiological and behavioural evidence to provide sufficient preclinical evidence to warrant clinical translation [256,269].

##### Graft Architecture and Rosette Formation

Organoid-derived retinal sheets frequently self-organize into rosettes, with photoreceptor inner and outer segments oriented toward a central lumen rather than the host RPE [258,270]. Rosette formation has been reported in sheet grafts across multiple preclinical models and in initial human studies, especially when the sheets lack an inner nuclear layer [239,258,269,270]. Rosettes can separate outer segments from the host RPE, complicating host–graft connectivity [269]. It has been shown that altering cellular composition, such as depleting bipolar cells, and using scaffold- and hydrogel-supported retinal sheet delivery can improve host-graft connectivity [271,272,273,274].

##### Dependence on the Host Retinal Pigmented Epithelium

Functional photoreceptors require apposition to metabolically competent RPE for outer-segment phagocytosis and 11-cis-retinal regeneration. Grafts may not achieve this, particularly when rosette formation directs outer segments away from the graft surface, and inefficient visual pigment recycling has been proposed to explain the reduced amplitude and temporal resolution of graft-associated responses [269]. Secondary RPE atrophy in advanced RP means that intact host RPE cannot be assumed. The evidence is not uniform, however: hESC-derived sheets transplanted into RCS rats, which have defective RPE phagocytosis, still developed outer-segment-like structures and were associated with improved visual function [270]. Combined RPE–photoreceptor transplantation has shown greater structural or functional preservation than single-cell approaches in some rodent models [275,276], although layered co-grafts appeared primarily neuroprotective rather than functionally restorative [277]. Whether combined photoreceptor–RPE transplantation is necessary, and for which patients, remains uncertain.

##### Retinal Remodeling

In RP, photoreceptor loss deafferents the neural retina and initiates retinal remodelling, a progressive, negative neural plasticity that reorganizes the surviving inner retina, which is not a passive scaffold awaiting retinal replacement [278,279]. It proceeds in 3 stages that reflect (1) photoreceptor stress, (2) photoreceptor loss, and (3) widespread rewiring and cell death [278]. Bipolar cells are affected first because they lose photoreceptor input and switch their glutamate-receptor expression, favouring a suppressed, or OFF, phenotype [279]. At the same time, Müller glia react most extensively, becoming hypertrophic and forming a distal glial seal that walls off the inner retina from the RPE and choroid, with subsequent metabolic collapse [278,279]. Horizontal and amacrine cells retract their normal processes and sprout aberrant neurites, forming ectopic contacts and tangled microneuromas that carry active but disordered synapses [278,279].

Transplantation assumes that grafted cells integrate and reconnect with existing synaptic pathways, both of which remodelling largely impairs. A study by Santos-Ferreira and colleagues was integral in showing that transplanted cells in mouse models of RP do not integrate at the previously assumed rate; rather, much of what was interpreted as integration was, in fact, cytoplasmic transfer of donor material to host cells [243]. Another study by Barber and colleagues showed lower integration rates at sites of glial scarring and that enzymatically breaking down the glial scar’s extracellular matrix significantly increased integration in mouse models of RP [280]. Another key mouse study showed that even when donor cells integrate, the host circuit they drive must be reprogrammed, as bipolar cells have shifted to an OFF state; so successful integration does not guarantee visual signalling [281]. Finally, because these barriers intensify in more advanced stages of RP, successful integration and restoration of signalling is likely to occur only in early RP [278]. Thus, retinal remodelling largely limits functional integration, and considerable work remains to develop effective transplantation strategies that can overcome it.

##### Immune Rejection, Tumorigenicity, and Manufacturing

Reprogramming and prolonged culture of hiPSCs may introduce genomic instability, leading to acquired mutations and abnormal differentiation [282]. Tumorigenic potential is not unique to either source, as both hESC and hiPSC-derived cells can contain residual undifferentiated cells or acquire genomic abnormalities during the culture process [240,282]. The inherent risk of tumorigenesis thus necessitates rigorous safety and quality-control measures.

Allogeneic hESC- or hiPSC-derived products can be generated from standardized cell banks and are scalable, but require HLA matching or immunosuppression to reduce rejection [240,282]. The generation of autologous products is not cost-effective and requires correction of causal RP mutations [283]. However, it can address some challenges associated with immune rejection.

### 7.2. Cell-Based Neuroprotection

#### 7.2.1. Bone Marrow-Derived Cell Therapies

Researchers primarily investigate bone marrow-derived cells as cell-based neuroprotective therapies. The term encompasses several distinct products—bone marrow mononuclear cells (BMMCs), mesenchymal stromal cells (MSCs), and CD34+ hematopoietic stem cells—which differ in isolation method, mechanism, and safety profile. Subretinal transplantation of RPE-like cells differentiated from bone-marrow was successful in animal models; however, it did not restore visual function or retinal morphology [284]. Therefore, the therapeutic rationale for these products is predominantly paracrine, trophic, immunomodulatory, and vascular.

MSCs exert neuroprotective and immunomodulatory effects largely through paracrine signalling [285,286,287]. In photoreceptor-degeneration models, intravitreally injected lineage-negative bone marrow hematopoietic cells were associated with vascular stabilization and reduced retinal degeneration, supporting an indirect supportive mechanism rather than retinal-cell replacement [43]. Clinical evidence is limited and heterogeneous. A report of intravitreal autologous BMMC administration in RP improved patient-reported quality of life but did not establish objective structural or functional efficacy [288]. A nonrandomized phase I study of intravitreal autologous MSCs in 14 patients with RP found no improvements in vision, but reported no disease progression and manageable adverse events [263]. Another single-arm phase I study of intravitreal autologous CD34+ cells in 7 patients with RP, reported no treatment-related intraocular inflammation or cellular hyperproliferation [260]. The investigators noted that the procedure was safe and tolerable, with future studies required to determine potential efficacy [260,289]. These studies were largely proof-of-concept investigations designed to establish safety rather than efficacy, and future studies are warranted to establish therapeutic effect.

Severe complications have been reported following intraocular administration of mesenchymal- and adipose-derived cell preparations in commercial clinics, including proliferative vitreoretinopathy, tractional and rhegmatogenous retinal detachment, and irreversible vision loss [290,291]. These complications may result, in part, from myofibroblastic transdifferentiation of intravitreally delivered cells, leading to epiretinal membrane formation and vitreoretinal traction [292]. Accordingly, standardized manufacturing, regulatory oversight, and further clinical trials are required to establish bone marrow-derived therapies for RP.

#### 7.2.2. Retinal Progenitor Cell-Based Therapies

Retinal progenitor cells (RPCs) are lineage-restricted multipotent progenitors, rather than pluripotent stem cells, that generate retinal cell types during development. Researchers typically isolate human RPCs from the fetal retina during the second trimester and expand them in culture while retaining developmental marker expression [292]. Their therapeutic potential has been proposed to involve both limited retinal cell replacement and paracrine neuroprotection.

Early preclinical work established that transplanted RPCs survive in the host retina, express photoreceptor-associated markers, and preserve light-mediated behaviour in degenerative models [293]. Subsequent characterization of a clinical-grade human RPC product in the rat RP models confirmed donor-cell survival and photoreceptor preservation but found no evidence of donor differentiation into retinal cell types, favouring a trophic mechanism [294]. Retinal progenitors transplanted subretinally in *PDE6B*-deficient mice delayed degeneration and demonstrated signs of integration while improving electroretinographic and behavioural responses [295]. These cells were delivered at postnatal day 7, immediately before the onset of degeneration, supporting their potential as a preventative treatment [295]. Transplantation into advanced, degenerating eyes has been reported separately with evidence of synaptic repair and partial visual restoration [296]. These findings establish a trophic effect with therapeutic promise in preclinical models that warrants further characterization before effective clinical translation [267,268].

Intravitreal allogeneic human RPCs (jCell; famzeretcel) were evaluated in a prospective, multicenter, open-label, single-arm phase I/IIa trial in 28 adults with nonsyndromic RP at doses ranging from 0.5 to 3.0 million cells [261]. No graft rejection was reported, and injections were well tolerated with only mild adverse events, establishing the safety of the therapy [261]. However, a subsequent randomized, sham-controlled phase IIb trial found no significant improvement in BCVA (NCT03073733) [297]. Clinical trials of bone marrow-derived, mesenchymal, neural progenitor, and retinal progenitor cell therapies are summarized in Table 6.

#### 7.2.3. Limitations of Intravitreal Injections of Cell-Based Therapies

Intravitreal delivery deposits cells in the vitreous, so retinal penetration is poor [285]. As a result, cells retained in the vitreous are unlikely to migrate into the outer retina or replace lost photoreceptors, so any benefit would most plausibly result from diffusible trophic or immunomodulatory signals acting on the surviving host retina [285]. This interpretation is consistent with the absence of demonstrated donor differentiation in preclinical characterization [261,294]. RPCs and bone-marrow derived cells therefore occupy an intermediate position between retinal replacement and cell-based neuroprotection.

Overall, current stem-cell approaches have shown functional benefits in preclinical models, but reproducible functional benefit in patients remains to be established. This limited clinical benefit likely reflects several unresolved challenges, including mechanistic uncertainty over integration versus material transfer, difficulty forming functional synaptic connections with the host retina, delivery constraints, immunogenicity and unresolved manufacturing issues.

## 8. Delivery Platforms

A major challenge in any pharmacologic or genetic therapy is optimizing delivery of the agent. The eye is unique due to its accessibility and immune-privileged status [298]. Administration of personalized therapeutics is relatively straightforward compared to other organs, as therapies can be injected intravitreally or subretinally, bypassing the need for systemic administration. The eye contains natural anatomical barriers, predominantly the blood-retinal barrier, that sequester the retina from the immune system and prevent systemic leakage of personalized therapies [298]. Despite these advantages of the eye as a route for drug delivery, significant barriers in delivery remain. Predominantly, current delivery platforms are limited by their transduction efficacy, carrying capacity and immunogenicity [299].

### 8.1. Virus-Based Gene Delivery

Three main categories of viral-based gene delivery systems exist: adenoviruses (AdVs), AAVs, and lentiviruses (LVs) [300]. Advantages and limitations of these vectors are summarized in Table 7.

#### 8.1.1. Adenovirus

Adenoviruses (AdVs) are non-enveloped double-stranded DNA viruses with a high carrying capacity of up to 37 kb that do not integrate into the host genome [307]. More than 120 AdV subtypes exist, and AdV2 and AdV5 have been extensively studied and shown to transduce RPE cells, with mixed evidence of photoreceptor transduction [307]. Although the absence of integration reduces the risk of insertional mutagenesis, transgene expression in dividing cells may wane over time, limiting the durability of therapeutic effects [308]. As recently as 2022, AdVs were the most commonly used viral vector in clinical trials [309,310]. However, their use has since declined because of their high immunogenicity, which leads to immune-mediated vector clearance [309,311].

#### 8.1.2. Adeno-Associated Virus

Today, AAVs are the leading vector in ocular gene therapy because they can transduce various tissues and support long-term gene expression [309,312,313]. As recently as 2023, over 80% of ocular gene therapy trials employed AAV vectors [314]. AAVs are single-stranded DNA viruses of about 4.7 kb that rely on co-infection, predominantly with AdV, for viral replication [315]. Their reliance on other viruses for infection makes them prime candidates for therapeutic delivery of exogenous genes, as they carry a low risk of viral immunogenicity. AAVs have high transduction efficacy, allowing them to spread easily through the retina, and their ability to transduce various retinal cell types is a major advantage [123]. This property reflects the diversity of AAV serotypes [313]. For example, AAV2 has been shown to transduce RGCs efficiently but photoreceptors poorly [298,316,317,318]. Recombinant AAVs (rAAVs), or pseudotypes, are engineered by exchanging viral capsids of different AAV serotypes [319]. The resulting vector contains the capsid of one AAV and the genome of another. The resulting rAAVs have greater cellular specificity, with some designed to transfect specific retinal cells, including photoreceptors, RGCs, Müller cells, and the RPE [298]. AAVs primarily exist as intracellular episomes, but they can occasionally insert into the genome; this risk is reduced with rAAVs, limiting the risk of insertional mutagenesis [320]. AAVs also have lower immunogenicity than other viruses, allowing for sustained expression and repeated administration [298,321]. However, with repeated administration, AAV immunogenicity increases.

#### 8.1.3. Lentivirus

LVs are retroviral particles with single-stranded RNA genomes that integrate into the host genome. This integration allows LVs to have durable transgene expression after a single administration, but it also carries a risk of mutagenesis [299,322,323]. Initial LVs were derived from HIV-1; however, they showed poor ability to transduce photoreceptors [324,325]. More recent LVs are derived from equine anemia virus and have been shown to efficiently transduce photoreceptors [315,325,326]. LVs are ideal for diseases like Stargardt disease and Usher syndrome, because they have a larger cargo capacity and can carry genes up to 10 kb [325,326,327,328]. Despite their promise, concerns remain, including the risk of producing replication-competent or pathogenic LVs, immunogenicity, and insertional mutagenesis [309,329].

### 8.2. Lipid-Based Nanoparticles

Non-viral gene delivery systems, most notably Lipid-based nanoparticles (LBNPs), have emerged as alternatives to viral vectors. Key advantages of LBNPs compared with viral vectors include their negligible immunogenicity, malleable targetability, stability during storage, and easily scalable production [330]. This section will discuss the prominent LBNPs, including liposomes, niosomes, solid lipid nanoparticles (SLNs) and nanostructured lipid carriers (NLCs).

#### 8.2.1. Liposomes and Niosomes

Liposomes consist of one or more phospholipid bilayers that mimic the plasma membrane and enclose an aqueous compartment. This aqueous core provides an ideal environment for encapsulating hydrophilic substrates, such as nucleic acids. They are highly biocompatible, and their properties can be manipulated to improve targeted delivery to retinal cells [305,331,332]. Hangai and colleagues demonstrated that liposomes could transduce nearly all retinal layers in in vivo models with limited immunogenicity [333]. Furthermore, retinal cell-specific targeting can be enhanced using cell-specific gene promoters [330,334]. However, despite these promising results, liposomes have been limited by drug leakage, phospholipid oxidation, and lower stability compared to other nanoparticles [335].

Niosomes are highly similar to liposomes; however, they replace phospholipids with non-ionic surfactants [336]. They retain many liposome properties, including high biodegradability, biocompatibility, and low immunogenicity, while being cheaper to manufacture [335]. Recent advances have modified niosome properties, demonstrating that specific modifications can enhance transduction of specific retinal layers in preclinical models [337,338,339].

#### 8.2.2. Solid Lipid Nanoparticles and Nanostructured Lipid Carriers

Lipid-based nanoparticles include SLNs, which are the second most widely investigated class of LNPs after liposomes. SLNs are solid particles consisting of a lipid matrix stabilized by surfactants [340]. In the context of gene therapy, cationic SLNs have shown the most promising results, as their positively charges surfaces interact efficiently with negatively charged nucleic acids [330,341]. This positive charge is advantageous because it enhances stability and reduces drug mobility, prolonging drug release [335]. SLNs have been established in preclinical models as a viable option for delivery of plasmids to the retina [342,343,344].

NLCs are the second generation of SLNs designed to overcome their predecessor’s limitations. Researchers developed NLCs by adding a liquid lipid to the matrix, which distorts its structure and improves both stability and carrying capacity compared with SLNs [335,345]. One drawback is that the improved stability reduces drug expulsion, which can limit release in target cells [335,346]. Preclinical studies have established NLCs as a viable platform for delivering molecules to the retina [335,347].

### 8.3. Route of Delivery

Two principal routes deliver gene therapies to the retina, with both subretinal and IVIs offering distinct strengths and limitations. The choice largely involves a trade-off between transduction efficiency and surgical risk [348]. Subretinal injection places the vector directly between photoreceptors and the RPE, achieving efficient transduction of the degenerating retinal layer affected in RP. It also reduces the risk of immune reaction against the vector, as the subretinal space is immune-privileged [348]. However, the procedure requires vitrectomy and a deliberate, localized retinal detachment to form a subretinal bleb, carrying surgical risks including retinal thinning, retinal holes (including macular holes), and retinal detachment [348]. IVIs, by contrast, are simpler and less invasive, but the inner limiting membrane largely limits transduction of the outer retina and photoreceptors [348]. To address this limited transduction, researchers are engineering vectors with deeper transduction [349]. IVIs are also a safer, in-office procedure, posing less risk of retinal thinning and detachment. Currently, the only approved retinal gene therapy is administered by subretinal injections [67].

## 9. Comparison of Therapeutic Strategies

Therapeutic strategies are best viewed as complementary rather than competing, with the most appropriate approach largely determined by mutation mechanism and disease stage (Table 8). Mutation and mechanism dictate the modality class: loss-of-function alleles suit gene augmentation, whereas gain-of-function and dominant-negative alleles require suppression, editing, or RNA-based knockdown. Genotype-independent strategies, including gene-agnostic modifiers, neuroprotection, and cell replacement, apply across mutations but may be limited by disease stage. Disease stage governs feasibility; gene-editing strategies rely on the presence of viable photoreceptors and are thus suited to mild stages of RP. Neuroprotective strategies may be viable throughout the course of RP to halt progression, and cell replacement can be supportive in early stages; in later stages, it can attempt to replace photoreceptors, a process limited by viable, functional synapse formation. Finally, strategies vary in durability: RNA-based strategies such as ASOs or neuroprotective strategies may require multiple administrations, gene-editing therapies can provide long-term effects dependent on high transduction, and cell replacement strategies also aim to provide long-term effects. While these strategies remain largely preclinical, successful translation would give clinicians a diverse toolset to address photoreceptor degeneration in RP.

## 10. Conclusions and Future Perspectives

RP is highly genetically heterogeneous, with mutations in several key molecular pathways. While a “one-size-fits-all” treatment would be highly effective, many novel therapies that target genetic abnormalities are mutation-specific. These primarily include gene- and RNA-based therapies, which have shown promising preclinical results by directly targeting disease-driving genetic abnormalities. These approaches offer the potential for long-term correction and precise modulation of pathogenic mechanisms, but their applicability is limited to patients with defined mutations, and challenges remain in achieving efficient, safe delivery. Advancements in CRISPR technology, RNA design, and viral vectors continue to expand therapeutic potential, though concerns such as off-target effects and immune responses must be addressed before clinical translation. Neuroprotective agents have emerged as complementary approaches, aiming to preserve remaining photoreceptors and prevent degeneration by mitigating oxidative stress and cell death. Their appeal lies in their broader applicability across multiple genetic forms of RP and generally favourable safety profile. However, their ability to reduce disease progression is often limited, and sustained or repeated administration may be required to maintain therapeutic benefit. In contrast, stem cell therapies offer a potential avenue to replace lost photoreceptors and restore retinal architecture, regardless of the underlying pathogenic mutation. Preclinical studies have shown promising functional and structural integration, and some early clinical trials have established safety, with some evidence of partial functional improvement. However, challenges remain, predominantly in determining and controlling whether transplanted cells act through supportive effects in milder disease or through integration and synapse formation in advanced RP.

Looking forward, many promising RP therapies offer patients and clinicians hope. Many promising developments from preclinical models are now being translated into the clinic and may provide clinicians with new therapeutic options for patients with RP. However, these novel therapies are expected to be costly. Luxturna, the only approved ocular gene therapy, costs 850,000 USD for both eyes [350]. Thus, accessibility will depend on value-based pricing models, reimbursement frameworks, and healthcare infrastructure that can deliver these novel therapies. Furthermore, except for *RPE65*-directed gene augmentation, the approaches discussed in this review remain investigational, with many still in preclinical development. Long-term safety, efficacy and durability have not been established, and even approved therapies do not halt progression. Nevertheless, emerging innovations in RP management signal a paradigm shift toward disease-modifying, personalized intervention for RP, providing both hope to patients and clinicians.

## Figures and Tables

**Table 1:** Representative genes associated with R, grouped by inheritance pattern and affected molecular pathways. ^†^ Denotes genes also causing syndromic forms of RP. ^††^ Denotes genes that also cause other IRDs.

Primary Mode of Inheritance	Symbols	Gene	Function	Prevalence	Other Modes of Inheritance/Syndromes	References
Autosomal Dominant	*RHO*	*Rhodopsin*	G-protein-coupled receptor that initiates phototransduction	30–40% of adRP	arRP	[11,12]
	*PRPF31*	*Pre-mRNA processing factor 31*	Component of the spliceosome	6–11% of adRP	-	[13]
	*PRPF3*	*Pre-mRNA processing factor 3*		1–2% of adRP	-	[14,15]
	*PRPF8*	*Pre-mRNA processing factor 8*		0–3% adRP	-	[15]
	*PRPH2*	*Peripherin 2*	Photoreceptor-specific tetraspanin critical to development of rod and cone outer segments	-	arRP, Retinitis punctata albescens, LCA, choroidal dystrophy, macular dystrophy	[16,17]
	*RP1*	*Retinitis pigmentosa 1*	Ciliary structure and function	3–5% of adRP	arRP	[18]
	*NR2E3*	*Nuclear receptor subfamily 2 group E member 3 (NR2E3)*	Component of the photoreceptor transcription cascade in retinal development	1% of adRP	arRP, Enhanced S-cone syndrome	[19,20]
	*snRNP200*	*Small nuclear ribonucleoprotein U5 subunit 200 (snRNP200)*	Component of the spliceosome	4–27% of adRP	-	[21,22,23]
	*CRX*	*Cone-rod homeobox*	Transcription factor regulating photoreceptor development	<1% of adRP	Leber congenital amaurosis	[24,25]
Autosomal recessive	*PDE6A*	*Phosphodiesterase 6A*	Component of the rod phototransduction cascade	2–4% of arRP	-	[26]
	*PDE6B*	*Phosphodiesterase 6B*	Component of the rod phototransduction cascade	4% of arRP	Night blindness (autosomal dominant)	[26]
	*CNGA1*	*Cyclic nucleotide-gated channel alpha 1*	Component of the rod phototransduction cascade	2–8% of arRP	-	[27]
	*ABCA4* ^††^	*ATP-binding cassette subfamily A member 4*	Cleanses metabolites of the visual cycle. Dysfunction is toxic to RPE and photoreceptors.	Approximately 16% of arRP	Stargardt disease, Cone-rod dystrophy, retinal dystrophy, Fundus flavimaculatus	[4,28]
	*RPE65* ^††^	*Retinal pigment epithelium-specific 65 kDa protein*	Component of the visual cycle in the RPE	0.6–6% of arRP	LCA, adRP	[29]
	*CRB1*	*Crumbs cell polarity complex component 1*	Involved in photoreceptor development	0–6.5% of arRP	LCA, Pigmented paravenous chorioretinal atrophy	[30]
	*USH2A* ^†^	*Usherin*	Transmembrane protein found in photoreceptors involved in retinal maintenance	8–19% of arRP	Usher Syndrome	[31,32]
X-linked Retinitis Pigmentosa	*RPGR*	*Retinitis pigmentosa GTPase regulator*	Localizes to outer segments of photoreceptors and is involved in cilia transport	75–91% of XLRP	Cone-rod dystrophy, Macular degeneration	[33,34]
	*RP2*	*Retinitis pigmentosa 2*	Role in trafficking lipidated proteins to the outer segment of photoreceptors	5–20% of XLRP	-	[35]

**Table 3:** Clinical Trials of CRISPR-Based Gene Editing Treatments for Retinitis Pigmentosa.

Trial ID (Start–End Year)	Trial Phase	Target Gene	Intervention	Primary Outcome
NCT05805007 (2023–2026)	I (Unknown)	*RHO*	Subretinal ZVS203e (rAAV8-CRISPR/Cas9)	Incidence of TEAEs
NCT06952842 (2025–2030)	I/II (Recruiting)	*RHO*	Subretinal ZVR203e (rAAV8-CRISPR/Cas9)	Incidence of TEAEs and change in BCVA

**Table 5:** Summary of clinical trials of Neuroprotective therapies for retinitis pigmentosa.

Trial ID (Start–End Year)	Trial Phase	Therapeutic Approach	Intervention	Primary Outcome	Findings	References
NCT05537220 (2023–2029)	III (Active)	Antioxidant therapy	Oral N-acetylcysteine	Progressive change in ellipsoid zone width	-	-
NCT03063021 (2017–2019) NCT03999021 (2019–2027)	I (Completed)			Tolerability and incidence of TEAEs	Results Unpublished	-
NCT04864496 (2021–2022)	II (Unknown)			Change in BCVA	-	-
NCT00447980 (2007–2010) NCT00447993 (2007–2009)	II/III (Completed)	Neurotrophic agent	Intraocular CNTF implant (Human NTC-501)	Change in Visual Field and BCVA	The treatment did not confer any visual improvements	[171,210]
NCT01530659 (2012–2019)	II (Completed)			Mean change in cone spacing	Results Unpublished	-
NCT02110225 (2014–2015)	Ib/II (Completed)	Neurotrophic agent	Topical rhNGF Drops	Tolerability, incidence of TEAEs and change in BCVA	Results Unpublished	-
NCT02609165 (2015–2017)	II (Completed)			Change in macular thickness	Results Unpublished	-

**Table 6:** Summary of stem cell therapy clinical trials for RP.

Trial ID (Start–End Year)	Trial Phase (Status)	Intervention	Primary Outcome	Published Findings	References
NCT02709876 (2014–2021)	I/II (Unknown)	Intravitreal BMSC (CD34+/CD133+/CD271+)	Change in VA	-	-
NCT05413148 (2022–2023)	II/III (Unknown)	Subtenon UC-MSC	Change in BCVA and visual field	-	-
NCT04284293 (2021–2026)	I/IIa (Active)	Subretinal hNPC	Incidence of TEAEs and safety	-	-
NCT04925687 (2021–2023)	I (Completed)	Intravitreal BMSC (CD34+)	Incidence of TEAEs	The therapy was well tolerated, with only 1 adverse effect noted	[260]
NCT02464436 (2015–2023)	I/IIa (Unknown)	Subretinal hRPC	Incidence of TEAEs	-	-
NCT03073733 (2017–2020)	IIb (Completed)	Intravitreal hRPC	Change in BCVA	Treatment was safe and resulted in improvements in BCVA	[261]
NCT06912633 (2025–2026)	II (Active)	Intravitreal hRPC	Incidence of TEAEs	-	-
NCT05712148 (2019–2022)	I/II (Completed)	Suprachoroidal MSC	Change in BCVA, visual field and ERG	Clinical improvement in BCVA, visual field, mfERG noted without any serious TEAEs	[262]
NCT05909488 (2025–2027)	I/II (Recruiting)	Peribulbar UC-MSC	Incidence of TEAEs	-	-
NCT06242379 (2024–2027)	I/II (Recruiting)	Intravitreal BM-MSC	Incidence of TEAEs	-	-
NCT01531348 (2012–2020)	I (Completed)	Intravitreal BM-MSC	Change in laser flare and cell measurements	Treatment was safe and showed potential effect with improvements in BCVA	[263]
NCT04763369 (2021–2022)	II (Unknown)	Subtenon UC-MSC	Incidence of TEAEs and change in BCVA	-	-
NCT04224207 (2019–2020)	III (Completed)	Subtenon Wharton Jelly (WJ)-derived MSC	Change in VA	Treatment was safe and led to improved BCVA	[264]
NCT05800301 (2019–2022)	III (Completed)		Fundus autofluorescence (FAF) surface area	Treatment can slow the progression of RP as measured on FAF	[265]
NCT05147701 (2022–2026)	I (Recruiting)	Subretinal UC-MSC	Incidence of TEAEs	-	-
NCT04604899 (2010–2022)	II (Completed)	Intravitreal hRPC	Incidence of TEAEs	Results Unpublished	-
NCT01736059 (2012–2026)	I (Active)	Intravitreal BM CD34+ Stem Cells	Incidence of TEAEs	Treatment was safe and well tolerated in patients with ischemic or degenerative retinal disease, including RP	[260]
NCT03944239 (2020–2021)	I (Active)	Subretinal hESC-derived RPE	Incidence of TEAEs	-	-
NCT03963154 (2019–2026)	I/II (Active)	Subretinal hESC-derived RPE	Incidence of TEAEs	Treatment was safe and resulted in anatomical stability of the graft confirmed by OCT	[260]
NCT06891885 (2025–2032)	I/IIa (Recruiting)	Subretinal iPSC-derived retinal sheets (DSP-3077)	Incidence of TEAEs	-	-
NCT03772938 (2018–2020)	I (Unknown)	Intravitreal BMSC	Incidence of TEAEs	-	-
NCT01920867 (2012–2020)	Interventional (Unknown)	BMSC	Change in BCVA	Treatment resulted in improved BCVA without any serious TEAEs	[266]
NCT06789445 (2025–2030)	I/II (Recruiting0	Subretinal iPSC (OpCT-001)	Incidence of TEAEs	-	-

**Table 7:** Summary of the advantages and limitations of vectors for gene delivery in ophthalmic applications.

Vector Category	Subtypes	Cargo Capacity	Advantages	Disadvantages	Integration/ Expression Profile	References
Viral-based vectors						
Adenovirus	E1/E3 depleted vectors	8 kb	High transduction; Reduced immunogenicity; Broad tropism profiles; Low risk of insertional mutagenesis	Strong immune response; transient expression; inflammatory potential in retina. Effects can wane in dividing cells	Non-integrating; transient expression	[299]
	E1 to E4 depleted vectors	12 kb			Non-integrating; transient expression	[299]
	High-capacity adenovirus vectors	36 kb			Non-integrating; Initially high but transient	[299]
Adeno-associated viruses	AAV2, AAV8	4.5 kb	Low immunogenicity; non-pathogenic; long-term expression in post-mitotic cells; broad retinal tropism; well-established in clinical trials	Small packaging size; potential mild immune responses; episomal, so dividing cells lose expression	Non-integrating; stable in post-mitotic cells; episomal persistence	[301]
Lentivirus	HIV-1	8–10 kb	Integrates into host genome for stable long-term expression; carry larger transgenes than AAV; infects dividing and non-dividing cells	Risk of insertional mutagenesis (e.g., myelodysplastic syndrome and myeloid leukemia); lower transduction efficiency in photoreceptors	Integrating; stable expression in dividing and non-dividing cells	[302,303]
Nonviral-based vectors						
Liposome	Cationic or neutral liposomes	>10 kb	Low immunogenicity; can carry large DNA/RNA constructs; relatively simple production;	Lower transfection efficiency compared with viral vectors; transient expression; limited tissue penetration	Non-integrating; transient expression	[304,305]
Solid lipid nanoparticles		>10 kb	Improved stability compared with liposomes; protect nucleic acids from degradation;	Lower gene delivery efficiency than viral vectors; high possibility of substrate expulsion after polymeric transition	Non-integrating; transient expression	[306]

**Table 8:** Comparison of Therapeutic Approaches.

Therapeutic Approach	Genotype-Specific/Agnostic	Optimal Disease Stage	Main Route	Major Advantage	Major Limitation	Clinical Stage
Gene augmentation	Genotype-specific	Early–mid (needs viable PRs)	Subretinal	Durable, single dose for Loss-of function mutations	RPE toxicity; does not halt degeneration	Clinical Trials; 1 FDA-approved therapy
Gene editing	Genotype-specific	Early–mid	Subretinal	Mutation-specific correction	Off-target; irreversible; requirement of large vectors	Preclinical/Phase I-II Clinical Trials
RNA-based Therapy	Genotype-specific	Early–mid	Subretinal/IVI	Reversible; no genome change	Transient—repeat dosing	Preclinical/Phase I-III Clinical Trials
Gene-agnostic modifiers	Gene-agnostic	Any	Subretinal	Generalized, mutation-independent treatment	Does not address the underlying cause	Preclinical/Phase I-III Clinical Trials
Neuroprotection/antioxidant	Gene-agnostic	Any	Oral, IVI	Mutation-independent	Does not address the underlying cause	Preclinical/Phase I-III Clinical Trials
Cell-death pathway inhibitors	Gene-agnostic	Any	Subretinal	IVI, subretinal		Preclinical
Stem-cell/cell replacement	Gene-agnostic	Advanced (PR depleted)	Subretinal	Potential to replace lost cells	Barriers preventing formation of functional synapses	Phase I-III Clinical Trials

## Data Availability

No new data were created or analyzed in this study. Data sharing is not applicable.
